# The RNA-binding protein KSRP aggravates malignant progression of clear cell renal cell carcinoma through transcriptional inhibition and post-transcriptional destabilization of the NEDD4L ubiquitin ligase

**DOI:** 10.1186/s12929-023-00949-9

**Published:** 2023-08-14

**Authors:** Yi-Chieh Yang, Yung-Wei Lin, Wei-Jiunn Lee, Feng-Ru Lai, Kuo-Hao Ho, Chih-Ying Chu, Kuo-Tai Hua, Ji-Qing Chen, Min-Che Tung, Michael Hsiao, Yu-Ching Wen, Ming-Hsien Chien

**Affiliations:** 1 Department of Medical Research, Tungs’ Taichung MetroHarbor Hospital, Taichung, Taiwan; 2 Graduate Institute of Clinical Medicine, College of Medicine, Taipei Medical University, 250 Wu Hsing Street, Taipei, 11031 Taiwan; 3 International Master/PhD Program in Medicine, College of Medicine, Taipei Medical University, Taipei, Taiwan; 4 Department of Urology, Wan Fang Hospital, Taipei Medical University, 111, Section 3, Hsing Long Road, Taipei, 11696 Taiwan; 5 Department of Urology, School of Medicine, College of Medicine and TMU Research Center of Urology and Kidney (TMU-RCUK), Taipei Medical University, Taipei, Taiwan; 6 Department of Medical Education and Research, Wan Fang Hospital, Taipei Medical University, Taipei, Taiwan; 7 Graduate Institute of Toxicology, College of Medicine, National Taiwan University, Taipei, Taiwan; 8 Department of Cancer Biology, Geisel School of Medicine at Dartmouth, Lebanon, NH USA; 9 Department of Surgery, Tungs’ Taichung Metro Harbor Hospital, Taichung, Taiwan; 10 Genomics Research Center, Academia Sinica, Taipei, Taiwan; 11 TMU Research Center of Cancer Translational Medicine, Taipei Medical University, Taipei, Taiwan; 12 Pulmonary Research Center, Wan Fang Hospital, Taipei Medical University, Taipei, Taiwan; 13 Traditional Herbal Medicine Research Center, Taipei Medical University Hospital, Taipei, Taiwan

**Keywords:** ccRCC, Progression, KSRP, NEDD4L, EMT

## Abstract

**Background:**

KH-type splicing regulatory protein (KHSRP, also called KSRP), a versatile RNA-binding protein, plays a critical role in various physiological and pathological conditions through modulating gene expressions at multiple levels. However, the role of KSRP in clear cell renal cell carcinoma (ccRCC) remains poorly understood.

**Methods:**

KSRP expression was detected by a ccRCC tissue microarray and evaluated by an in silico analysis. Cell loss-of-function and gain-of-function, colony-formation, anoikis, and transwell assays, and an orthotopic bioluminescent xenograft model were conducted to determine the functional role of KRSP in ccRCC progression. Micro (mi)RNA and complementary (c)DNA microarrays were used to identify downstream targets of KSRP. Western blotting, quantitative real-time polymerase chain reaction, and promoter- and 3-untranslated region (3'UTR)-luciferase reporter assays were employed to validate the underlying mechanisms of KSRP which aggravate progression of ccRCC.

**Results:**

Our results showed that dysregulated high levels of KSRP were correlated with advanced clinical stages, larger tumor sizes, recurrence, and poor prognoses of ccRCC. Neural precursor cell-expressed developmentally downregulated 4 like (NEDD4L) was identified as a novel target of KSRP, which can reverse the protumorigenic and prometastatic characteristics as well as epithelial-mesenchymal transition (EMT) promotion by KSRP in vitro and in vivo. Molecular studies revealed that KSRP can decrease NEDD4L messenger (m)RNA stability via inducing mir-629-5p upregulation and directly targeting the AU-rich elements (AREs) of the 3’UTR. Moreover, KSRP was shown to transcriptionally suppress NEDD4L via inducing the transcriptional repressor, Wilm's tumor 1 (WT1). In the clinic, ccRCC samples revealed a positive correlation between KSRP and mesenchymal-related genes, and patients expressing high KSRP and low NEDD4L had the worst prognoses.

**Conclusion:**

The current findings unveil novel mechanisms of KSRP which promote malignant progression of ccRCC through transcriptional inhibition and post-transcriptional destabilization of NEDD4L transcripts. Targeting KSRP and its pathways may be a novel pharmaceutical intervention for ccRCC.

**Supplementary Information:**

The online version contains supplementary material available at 10.1186/s12929-023-00949-9.

## Background

Renal cell carcinoma (RCC) is one of the most common lethal urologic cancers, and its incidence has increased year by year [1]. Based on a variety of histologic and molecular subtypes, RCC can mainly be distinguished into three different groups, including clear cell RCC (ccRCC), papillary type RCC (pRCC), and chromophobe RCC (chRCC). Among these, ccRCC is the most common and aggressive subtype which accounts for 75–80% of RCC cases [2]. Due to it being clinically silent and relatively difficult to diagnose, almost one-third of ccRCC patients have metastatic dissemination at diagnosis, and nearly half of all patients die from their disease [3]. From the beginning of cytokine treatment to targeted therapy and immune checkpoint blockade, therapeutic strategies for this deadly stage have changed in recent years, but these treatment strategies are still insufficient, and advanced ccRCC remains a lethal disease [4, 5]. Thus, discovering novel molecular insights, biomarkers, and related treatment targets would have great clinical impacts for managing ccRCC patients.

KH-type splicing regulatory protein (KHSRP, also known as KSRP or FUBP2) is a multifunctional RNA-binding protein (RBP) involved in transcriptional and post-transcriptional regulation of gene expressions including messenger (m)RNA transcription [6], pre-mRNA spicing [7], mRNA degradation [8], and micro (mi)RNA maturation [9]. KSRP-mediated gene expression in several innate and adaptive immune responses, DNA damage response, inflammatory diseases, tissue remodeling, and lipid metabolism has been extensively studied [6, 10]. Recently, accumulating evidence indicates that KSRP may act in multiple steps of cancer development through regulating its targeted mRNAs and miRNAs, but conflicting roles of KSRP were reported in different cancer subtypes. For example, KSRP was reported to suppress motility and to be correlated with favorable prognoses in brain tumors and non-small cell lung cancer (NSCLC) [11, 12]. The antimetastatic effect of KSRP in NSCLC was attributed to induction of maturation of microRNA (miR)-23a to mediate early growth responsive gene 3 (EGR3) mRNA degradation. In contrast, KSRP was shown to promote the growth or invasion of esophageal squamous cell carcinoma and melanomas, respectively through enhancing the maturation of miRNAs such as miR-21, miR-130b, and miR-301 [13] and inducing destabilization of killin mRNA [14]. Although the inconsistent roles of KSRP in the development of several cancers have been described, to the best of our knowledge, information concerning the relationship between KSRP and ccRCC, including the function, molecular mechanisms, and clinical potential is still unknown.

This study attempted to perform a global view of KSRP-targeted genes to comprehensively understand its role in ccRCC progression. Herein, we examined the effects of KSRP on ccRCC progression and indicated that silencing KSRP impairs ccRCC cell growth and metastasis in vitro and in vivo. Combining information from The Cancer Genome Atlas (TCGA)-Kidney Renal Clear Cell Carcinoma (KIRC) database with the results of miRNA and complementary (c)DNA microarrays, we identified the E3 ubiquitin ligase, neural precursor cell-expressed developmentally downregulated 4 like (NEDD4L), as a novel target of KSRP which can reverse KSRP-promoted ccRCC progression in vitro and in vivo. Mechanistically, upregulation of miR-629-5p and targeting of the AU-rich element (ARE) site within the 3’-untranslated region (3’UTR) of NEDD4L are critical for KSRP-induced NEDD4L mRNA destabilization. Induction of the transcriptional repressor, Wilms’ tumor 1 (WT1), was also crucial for KSRP-mediated transcriptional inhibition of NEDD4L mRNA. These findings may unveil a novel role of the KSRP-NEDD4L regulatory axis in ccRCC progression and present potential biomarkers or targets for predicting and treating ccRCC.

## Methods

### Data collection from bioinformatics analyses

The miRNA and mRNA sequencing data of TCGA-KIRC patients were retrieved from UCSC Xena (https://xena.ucsc.edu/). GSE15641 containing RCC tumors and normal renal tissues was obtained from Gene Expression Omnibus (GEO) datasets. E-MTAB-6692 and E-MTAB-1980 were obtained from the European Bioinformatics Institute (https://www.ebi.ac.uk/). For the miRNA sequencing analysis from TCGA-KIRC, miRNAs with reads per million mapped reads (RPM) of > 1 in more than 90% of patients were retained for analysis. Differentially expressed miRNAs between tumor and normal tissues in KIRC were identified using the limma package. miRNAs with a fold change (FC) of > 2 and a false discovery rate (FDR) of < 0.05 were considered significantly upregulated in tumor tissues. For TCGA RNA sequencing data, gene expressions were normalized by fragments per kilobase per million (FPKM) and log2-transformed. Microarray data of E-MTAB-6692 and E-MTAB-1980 were normalized by robust multichip averages (RMAs).

### ccRCC specimens and immunohistochemical (IHC) staining

An established human RCC tissue microarray (TMA) (BC07115a; US Biomax, Rockville, MD, USA) containing 79 ccRCC tissues and 10 normal kidney tissues was used to detect KSRP protein expression levels by IHC staining as previously described [15]. Briefly, paraffin-embedded ccRCC tissue sections were deparaffinized using a xylene solution and rehydrated by gradient ethanol concentrations. Antigen retrieval was then performed by boiling the deparaffinized sections in 0.1 M citric acid buffer, followed by incubation with a corresponding primary antibody at 4 °C overnight. Next, a secondary antibody was incubated with the slides at room temperature for 1 h, and signal development was performed with a diaminobenzidine (DAB) kit (Boster, Wuhan, China).

### Antibodies and reagents

The antibodies we used were as follows: NEDD4L (#4013), p-Akt (#9271), Akt (#9272), E-cadherin (#3195), vimentin (#5741), N-cadherin (#13116), Snail (#3879), and Slug (#9585; Cell Signaling Technology, Danvers, MA, USA); KSRP (#A302-021A; ThermoFisher Scientific, Waltham, MA, USA); WT1 (sc-7385), green fluorescent protein (GFP; sc-9996), Lamin A/C (sc-7292; Santa Cruz Biotechnology, Santa Cruz, CA, USA); β-actin (A5441; Sigma-Aldrich, St. Louis, MO, USA); and GAPDH (60004-1-Ig; Proteintech, Chicago, IL, USA). The reagents we used were as follows: dimethyl sulfoxide (DMSO) (#D2650), crystal violet (#C0775), MG132 (#M7449), actinomycin D (#A9415; Sigma-Aldrich); Lipofectamine 3000 transfection reagent (#L3000015), and total RNA extraction TRIzol reagent (#15596026; Invitrogen, Grand Island, NY, USA).

### Cell lines and cell culture

The Caki-1, 786-O, and ACHN human RCC cell lines were obtained from American Type Culture Collection (Manassas, VA, USA) and maintained in MEM (for Caki-1 and ACHN), while 786-O cells were cultured in RPMI1640 supplemented with 10% fetal bovine serum (Gibco BRL, Gaithersburg, MD, USA), 100 units/mL penicillin, 100 µg/mL streptomycin, and 1% glutamine at 37 °C in a 5% CO_2_ humidified atmosphere.

### Establishment of gene knockdown (KD) and overexpression of RCC cells

The pEGFP-KSRP and pLenti-NEDD4L vectors were respectively obtained from Dr. K.T. Hua (National Taiwan University, Taipei, Taiwan) and Applied Biological Materials (Richmond, BC, Canada). Short hairpin (sh)RNAs for KSRP, NEDD4L, and WT1 were purchased from the National RNAi Core Facility at Academic Sinica (Taipei, Taiwan). To overexpress KSRP, the pEGFP-KSRP vector was transfected into RCC cells using the Lipofectamine 3000 Transfection Reagent (Invitrogen) for 6 h according to the manufacturer’s protocols. To knock down the indicated genes (KSRP, NEDD4L, and WT1) or overexpress NEDD4L, we produced lentiviral particles expressing shRNAs of the indicated genes or pLenti-NEDD4L to infect RCC cells for 24 h according to the protocols from our previous study [16]. Targeting sequences of the shRNAs were as follow: KSRP shRNA-1, 5′-CGCCTACTACTCACACTACTA-3′; KSRP shRNA-2, 5′-GACTTCAATGACAGAAGAGTA-3′; NEDD4L shRNA, 5′-GTTGCTGGTCTGGCCGTATTT-3′; and WT1 shRNA, 5′-GCAGTGACAATTTATACCAAA-3′.

### Western blot assay

Protein samples were extracted by cold RIPA lysis buffer according to protocols from our previous study [15]. After determining the protein concentration by a bicinchoninic acid (BCA) assay (ThermoFisher Scientific), equal amounts of protein (20–40 µg) were separated by sodium dodecylsulfate polyacrylamide gel electrophoresis (SDS-PAGE) and transferred onto polyvinylidene difluoride (PVDF) membranes (Bio-Rad, Hercules, CA, USA). Separated proteins were detected with corresponding antibodies followed by an enhanced chemiluminescence (ECL) system (Pierce Biotechnology, Rockford, IL, USA).

### Plate colony-forming assay

ccRCC cells were plated into six-well plates at a density of 10^3^ cells/well and cultured for 7–10 days. Cells were then fixed with methanol followed by staining with 1% crystal violet to manually count the numbers of colonies using free ImageJ software (National Institutes of Health, Bethesda, MD, USA).

### Transwell invasion assay

An invasion assay was performed using 24-well transwell inserts (Corning Costar, Corning, NY, USA) according to protocols from our previous study [15]. Briefly, 5 × 10^4^ ccRCC cells were plated onto the top chamber coated with Matrigel (BD Biosciences, Bedford, MA, USA) and supplemented with serum-free medium. The lower chamber was supplemented with complete medium as a chemoattractant to attract cells. After 24 h of incubation, invaded cells on the lower surface were fixed with methanol and stained with 0.5% crystal violet. Numbers of stained cells were counted in at least three random microscopic fields (× 100).

### cDNA microarray and data analysis

Transcriptional profiling data were collected from a microarray analysis of Caki-1/shCtrl and Caki-1/shKSRP cells. cDNA hybridization was performed with Affymetrix human U133 2.0 plus arrays (ThermoFisher Scientific). Gene expression levels were normalized as log2 values using GeneSpring software (Agilent Technologies, Santa Clara, CA, USA), and the obtained list contained genes that were up- or downregulated with greater than twofold changes in response to KSRP-KD.

### miRNA OneArray^®^ and data analysis

To identify which miRNAs are regulated by KSRP, a high-throughput and specific miRNA microarray (human miRNA OneArray^®^ miRNA profiling chip) using Caki-1 cells with/without KSRP-KD was conducted by the Phalanx Biotech Group (Hsinchu, Taiwan). Each single sample was performed at least twice in terms of technical or biological replicates to reduce the variance. Statistical *p* values were calculated by Student's *t*-test, and fold changes were output to evaluate differentially expressed genes.

### Real-time reverse-transcription quantitative polymerase chain reaction (RT-qPCR)

Total RNA was extracted from KSRP-manipulated ccRCC cells using Trizol (ThermoFisher Scientific) and reverse-transcribed to cDNA using an iScript™ cDNA Synthesis Kit (Bio-Rad). The cDNA was further applied to the RT-qPCR using the TOOLS 2 × SYBR qPCR Mix kit (BIOTOOLS, Taipei, Taiwan), and GAPDH was set as the internal control. Primers used in the RT-qPCR are listed as follows: NEDD4L forward: 5′-AGCCCAATGGGTCAGAAATAA-3′; NEDD4L reverse: 5′-TCTGGACCCTGTTCACAAATC-3′; GAPDH forward: 5′-CTGGAGAAACCTGCCAAGTATGAT-3′; and GAPDH reverse: 5′-TTCTTACTCCTTGGAGGCCATGTA-3′. To detect miRNA expression levels, we used a TaqMan MicroRNA Assay kit (ThermoFisher Scientific) as previously described [17].

### Gene set enrichment analysis (GSEA) and pathway enrichment analysis

A GSEA was conducted to explore associations of KSRP, NEDD4L, and miR-629-5p expression with the epithelial-mesenchymal transition (EMT). The indicated genes were ranked based on the fold change between high expression (top 5%) and low expression (bottom 5%) groups in TCGA KIRC patients. A weighted Kolmogorov–Smirnov test was performed with 1000 permutations to calculate the normalized enrichment score (NES) and false discovery rate (FDR). The HALLMARK EMT pathway was used to conduct this analysis. To identify putative substrates ubiquitinated by NEDD4L, UbiBrowser (http://ubibrowser.ncpsb.org/ubibrowser/home/index) was used to predict substrates. Fifty-seven known and 2378 predicted substrates were obtained and applied to a pathway enrichment analysis to investigate which top signaling pathways involved these NEDD4L substrates. A hypergeometric test was performed to evaluate significant enrichment, and *p* values were adjusted by the Benjamini–Hochberg procedure.

### Zebrafish xenotransplantation model

Transgenic *Tg(fli1: EGFP)* zebrafish were used to establish a zebrafish xenograft model of human ccRCC. After being anesthetized with 0.1 mg/mL tricaine (Sigma-Aldrich), a microinjection of 400 of CM-DiI dye (ThermoFisher Scientific)-labeled Caki-1 and 786-O ccRCC cells expressing shKSRP or shCtrl was delivered to the zebrafish yolk sac. Tumor cell-bearing zebrafish were divided into shCtrl and shKSRP groups and maintained at 34 °C for a period of 4 days. Finally, the fluorescent signal was read to examine the distribution and metastasis of ccRCC cancer cells using a Zeiss Axiophot fluorescence microscope (Carl Zeiss Microimaging, Gottingen, Germany).

### In vivo orthotopic xenograft model

Orthotopic metastatic mouse models were performed according to protocols from our previous study [18]. We used 6–8-week-old male nonobese diabetic (NOD)-SCID mice to implant luciferase-tagged Caki-1 cells (10^6^) respectively expressing shKSRP, shNEDD4L, shKSRP/shNEDD4L, or a control vector into the renal capsule of the mice. After tumor cell implantation, we used a noninvasive bioluminescent imaging system, Xenogen IVIS-200 (Xenogen, Alameda, CA, USA), to monitor the tumor size and location once a week. After 5 weeks, all experimental mice were sacrificed, and luciferase activities in the excised organs (liver, pancreas, and lungs) were further determined using the IVIS-200 system. Primary tumor specimens were photographed, fixed, and sectioned for hematoxylin and eosin (H&E) and IHC staining. All animal experimental protocols were evaluated and approved by the Academia Sinica Institutional Animal Care and Utilization Committee (19-12-1388).

### Subcellular fractionation

Cytoplasmic and nuclear fractions were prepared using an NE-PER Cytoplasmic and Nuclear Protein extraction kit (ThermoFisher Scientific). Each fraction was resolved by SDS-PAGE and probed for WT1, KSRP, and NEDD4L. Fraction purity was assessed by probing for GAPDH for the cytoplasm and lamin A/C for nuclei.

### Anoikis assay

ccRCC cells with with/without KSRP-KD were seeded in 12-well ultra-low-attachment surface plates (Corning Costar) for 24 h. Then, cells were collected and re-seeded into 96-well plates for 3 h to evaluate the cell viability by using a CCK-8 assay kit (Abcam, Cambridge, MA, USA) according to the manufacturer’s instructions.

### Analysis of RNA stability

ccRCC cells with/without KSRP-KD were treated with the transcriptional inhibitor, actinomycin D (5 µg/mL), for the indicated time periods. NEDD4L mRNA levels were detected by an RT-qPCR and normalized to GAPDH.

### Construction of NEDD4L-3′UTR reporter plasmids with different mutated ARE sites and a luciferase reporter assay

The wild-type (WT) NEDD4L-3′UTR reporter was first constructed using primer sets with the restriction enzyme cutting sites, F_SacI: AGAGCTCGAAACCTTTGAAGATTTA and R_XbaI: ATCTAGAGAAAGGATGTAAAAT. The PCR product was then inserted into a pmirGLO Vector (Promega, Madison, WI, USA). Mutations were introduced into the ARE of the 3’UTR using a QuikChange Lightning Site-Directed Mutagenesis Kit (Agilent Technologies). We mutated the central U of AUUUA or AUUUUA to C using the following primers: Mut_1 forward 5′-TCAGGAGGACTTAATGCTAT**C**TATGTTGTGCCTCTGCAGGC-3′ and Mut_1 reverse 5′-GCCTGCAGAGGCACAACATAGATAGCATTAAGTCCTCCTGA-3′; and Mut_2 forward 5′-GCAAAGCCCTTAATAAATAT**CC**TACATCCTTTCTAATGACAA-3’ and Mut_2 reverse 5′-TTGTCATTAGAAAGGATGTAGGATATTTATTAAGGGCTTTGC-3′.ccRCC cells with/without KSRP-KD were seeded in 6-cm dishes and transfected with the WT NEDD4L 3′UTR or mutant plasmids by Lipofectamine 3000 for 48 h. Cell lysates were harvested and firefly luciferase activity was determined using a Dual-Luciferase Reporter Assay kit (Promega) with a SpectraMax L luminescence microplate reader (Molecular Devices, San Jose, CA, USA). The relative luciferase activity was calculated according to the formula: relative luciferase activity (fold) = (luciferase activity of sample with KSRP-KD)/(luciferase activity of control sample).

### Construction of NEDD4L promoter/reporter plasmids and a luciferase reporter assay

The human NEDD4L promoter region was cloned into pEZX-LvPG02 to obtain pEZX-LvPG02-NEDD4L and purchased from GeneCopoeia (Rockville, MD, USA). Three different promoter fragments were obtained using specific primers and subcloned into the original vector. ccRCC cells with/without KSRP-KD were seeded at 4 × 10^5^ cells in 6-cm dishes and cotransfected with NEDD4L promoter constructs (WT, F1, F2, or F3). To measure promoter activity, medium was harvested, and a Gaussia luciferase activity was determined using a Secrete-Pair™ Gaussia Luciferase Assay kit (GeneCopoeia). Cell lysates were harvested, and Renilla luciferase activity was determined as the internal control by a luciferase assay kit (Promega).

### Statistical analysis

Values of the in vitro and in vivo studies are presented as the mean ± standard deviation (SD). Data were analyzed using Student’s *t*-test when two groups were compared. In the analyses of clinical data, correlation analyses of indicated genes and miR-629-5p expression were evaluated by Pearson correlations. Associations of gene expressions with overall survival (OS) and disease-specific survival (DSS) were evaluated by a log-rank test. For three-group comparisons, a pairwise log-rank test was performed to compare each group. The expression cutoff to define high or low expression groups was determined based on the minimum log-rank test *p* value. Gene expressions from paired tumor/normal tissues were compared by a paired *t*-test. To compare gene expressions in different clinical features, a Pearson’s Chi-squared test or Wilcoxon signed-rank test was performed. For gene expression differences among normal tissues, metastatic, and non-metastatic tumor tissues, a Kruskal–Wallis test with Dunn's post-hoc test was carried out.

## Results

### KSRP is upregulated in ccRCC specimens and is correlated with tumor progression and a poor prognosis

**Fig. 1:**
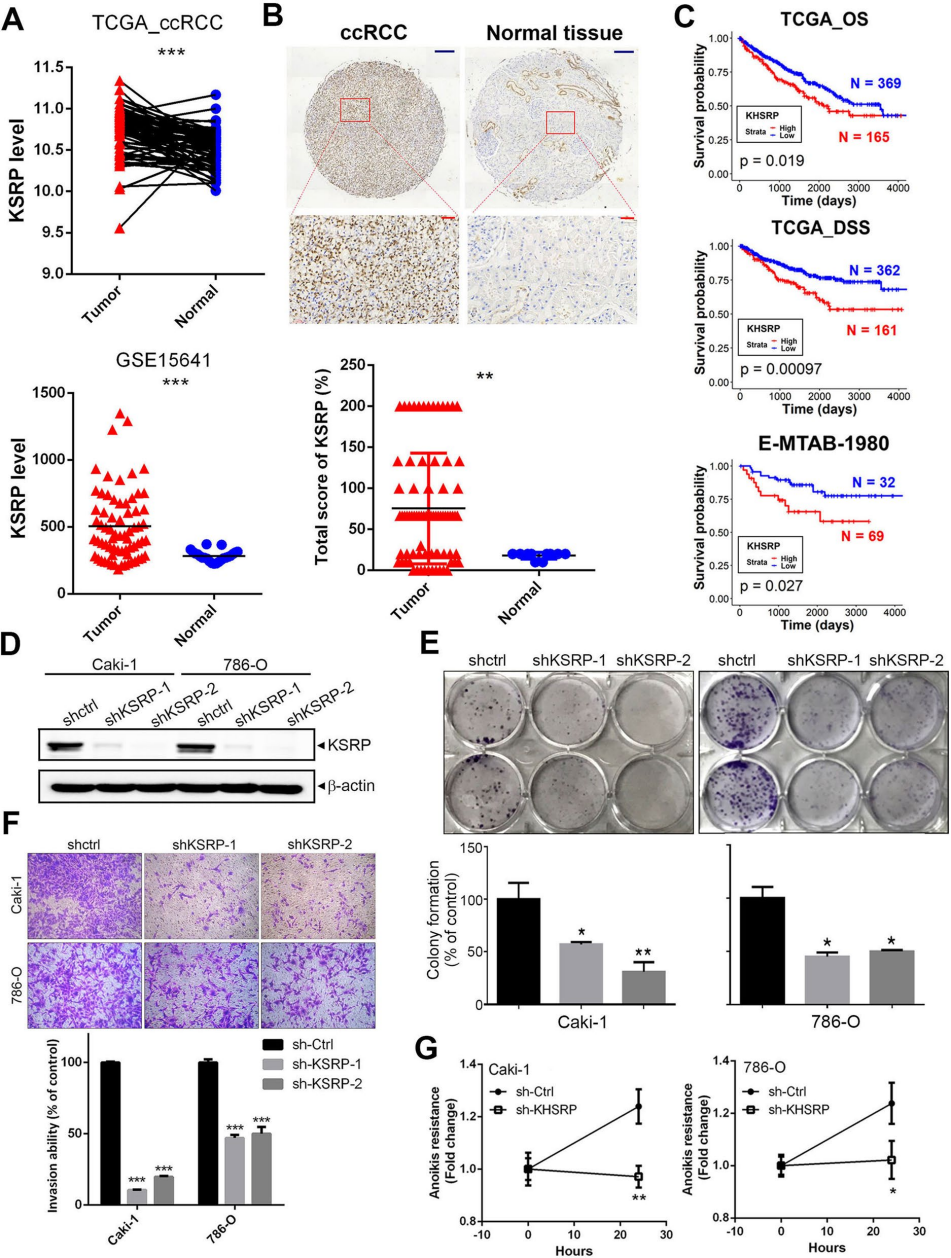
KH-type splicing regulatory protein (KSRP) expression is correlated with a poor prognosis of clear cell renal cell carcinoma (ccRCC) patients and induces clonogenicity, invasion, and anoikis resistance of ccRCC cells. **A** Upper panel, KSRP expression was analyzed in 72 matched ccRCC tissues and their corresponding normal tissues using data from TCGA. Lower panel, Plot depicting expression levels of KSRP in ccRCC specimens (*n* = 69) and normal tissue samples (*n* = 23). The plot was made using GSE15641 from GEO. **B** Upper panel, IHC staining of KSRP in ccRCC specimens and normal tissue samples. Lower panel, IHC summed scores were applied to determine KSRP expression. Scale bars of black color and red color are 100 and 30 μM, respectively. **C** Kaplan–Meier analysis of overall survival (OS) and disease-specific survival (DSS) rates in patients with ccRCC presenting with high or low KSRP expression using data from TCGA or E-MTAB-1980. **D** Knockdown efficiencies of two KSRP shRNAs were determined by Western blotting in Caki-1 and 786-O cells. **E–G** The effects of KSRP*-*knockdown on the growth, invasion, and anoikis of Caki-1 and 786-O cells were respectively examined by colony formation (**E**), Matrigel invasion (**F**), and anoikis resistance (**G**) assays. Values are presented as the mean ± SD of three independent experiments. *p < 0.05; **p < 0.01; ***p < 0.001, compared to the control group

To explore the clinical relevance of KSRP in ccRCC, we first evaluated expression levels of the KSRP gene between tumor vs. noncancerous tissues in two independent databases of GEO and TCGA-KIRC. Significantly higher levels of KSRP transcripts were observed in tumors compared to normal tissues (GSE15641) (Fig. 1A, lower panel). No matter the primary and metastatic ccRCC tumors from TCGA-KIRC cohort all expressed higher KSRP transcripts than did normal tissues (Additional file 1: Fig. S1). Moreover, 72 tumor tissues and their corresponding matched normal tissues were further analyzed from the same cohort, and we found significantly higher KSRP transcripts in tumors than in adjacent normal tissues (Fig. 1A, upper panel). In addition to mRNA levels, KSRP protein levels were analyzed in another ccRCC TMA cohort, and significant upregulation of KSRP was observed in tumors compared to normal tissues (Fig. 1B). Next, a Chi-squared analysis was conducted to explore relationships between clinicopathologic features and KSRP expression in the TCGA-KIRC cohort. Table 1 shows that KSRP expression levels were significantly correlated with the tumor size (*p* = 0.02), clinical stage (*p* = 0.011), and tumor recurrence (*p* = 0.004) and had a trend of being associated with distal metastasis (*p* = 0.056). Furthermore, the resulting Kaplan–Meier (KM) plot showed that ccRCC patients from TCGA-KIRC and E-MTAB-1980 cohorts with higher KSRP expression all had worse OS or DSS than those with lower KSRP levels (Fig. 1C). Taken together, these clinical data imply that KSRP is a potential prognostic factor affecting the survival of ccRCC patients and may play a critical role in the progression of ccRCC.

**Table 1:** Correlation of clinical parameters and KH-type splicing regulatory protein (KSRP) in clear cell renal cell carcinoma (ccRCC)

Clinicopathological feature	N	KSRP expression, n (%)		
	534	Low	High	*p* value
		369 (69.1)	165 (30.9)	
T stage				
T1 + T2	343	253	90	***0.002***
T3 + T4	191	116	75	
N stage				
N0	240	163	77	0.654
N1	16	10	6	
M stage				
M0	422	297	125	0.056
M1	79	47	32	
Stage				
I + II	325	238	87	***0.011***
III + IV	207	130	77	
Recurrence				
No	414	299	115	***0.004***
Yes	109	63	46	

The significance of the statistical analysis is indicated by the bold and italic values of the *p*-value

We next evaluated the effect of KSRP expression on cell behaviors including cell growth and metastasis, two fundamental steps of tumor progression. First, lentiviral-based RNA-KD and -overexpression approaches were used to establish stable knockdown and overexpression of KSRP in Caki-1 and 786-O ccRCC cells (Fig. 1D; Additional file 1: Fig. S2A). We first examined the effect of KSRP on long-term growth (8–10 days) of Caki-1 and 786-O cells using a colony-formation assay and observed that knockdown of KSRP expression significantly suppressed cell clonogenicity (Fig. 1E). Invasion and anoikis resistance are key elements of cancer metastasis, and we found that knockdown of KSRP also remarkably reduced invasion and anoikis resistance in both ccRCC cell lines (Fig. 1F, G). In contrast to the anticancer effects of KSRP-KD on ccRCC cells, KSRP overexpression caused opposite effects (Additional file 1: Fig. S2B).

### NEDD4L is a potential downstream effector of KSRP and is correlated with a favorable prognosis of ccRCC patients

**Fig. 2:**
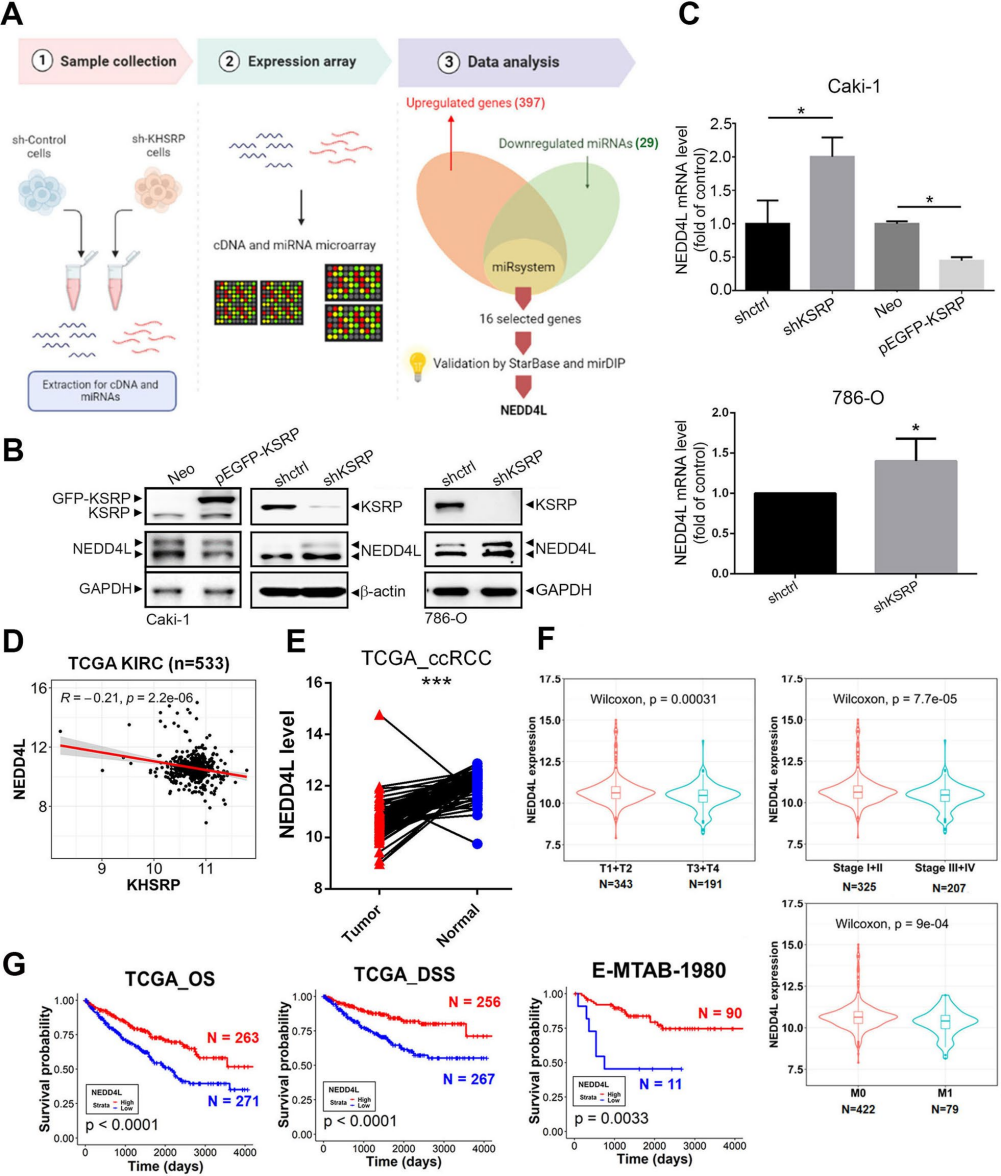
Neural precursor cell-expressed developmentally downregulated 4 like (NEDD4L) is negatively regulated by KH-type splicing regulatory protein (KSRP) and associated with a favorable prognosis in clear cell renal cell carcinoma (ccRCC). **A** Flowchart for integrating microRNA and cDNA microarrays regulated by KSRP and selection of NEDD4L as a potential KSRP-targeted gene. **B**, **C** Western blotting (**B**) and real-time qPCR (**C**) analyses of NEDD4L levels in ccRCC cells transfected with a KSRP shRNA, pEGFP-KSRP, or corresponding control vector. Values from (**C**) are presented as the mean ± SD of three independent experiments. *p < 0.05, compared to the control group. **D** Correlation analysis of the KIRC database (TCGA, PanCancer Atlas) using cBioPortal revealed negative correlations between mRNA levels of KSRP and NEDD4L. **E** NEDD4L expression was analyzed in 72 matched ccRCC tissues and their corresponding normal tissues using data from TCGA-KIRC. **F** NEDD4L expression levels obtained from TCGA-KIRC were compared according to the tumor size (T stages), clinical stages, and metastasis (M stages). Statistical significance was analyzed by a Wilcoxon signed-rank test. **G** Kaplan–Meier analysis of overall survival (OS) and disease-specific survival (DSS) rates in patients with ccRCC presenting with high or low expression of NEDD4L using data from TCGA and E-MTAB-1980

The ability of KSRP to modulate tumor progression was reported to be derived from its ability to regulate mRNA stability through inducing miRNA biogenesis or ARE-binding [6, 19]. In an attempt to elucidate this possibility in ccRCC, we performed miRNA (OneArray^®^ v7, Phalanx Biotech Group) and cDNA microarray analyses (Affymetrix human U133 2.0 plus) on Caki-1/shCtrl and Caki-1/shKSRP cells to explore potential miRNAs and genes regulated by KSRP (Fig. 2A). We obtained 29 downregulated miRNAs and 397 upregulated genes in KSRP-KD cells. Next, we input these 29 miRNAs into an miRNA target gene prediction program, miRSystem (http://mirsystem.cgm.ntu.edu.tw/), to predict potential genes which could be targeted by 29 of the KSRP-regulated miRNAs. Overlapping potential target genes by 29 miRNAs to 397 upregulated genes, we finally obtained 16 candidate genes (Tables 2 and 3). Furthermore, we verified these 16 genes using the MirDIP and Starbase websites and recorded predictions of 29 miRNA-targeted genes in Tables 2 and 3. Results from these two independent online resources all showed that an E3 ubiquitin ligase, the NEDD4L gene, was predicted to be targeted by most of the 29 KSRP-regulated miRNAs (Tables 2 and 3). Hence, we speculated that NEDD4L might be an important target of KSRP in ccRCC cells. NEDD4L was previously reported to weaken or promote the progression of various cancers by targeting different substrates [20]. To verify the relationship between NEDD4L and KSRP, we knocked down KSRP in Caki-1 and 786-O cells and found that KSRP-KD cells showed increased protein (Fig. 2B) and mRNA (Fig. 2C) expression levels of NEDD4L compared to control ccRCC cells. In contrast, overexpressing KSRP in Caki-1 cells reduced NEDD4L protein and mRNA expression levels compared to vector control cells (Fig. 2B, C). In addition to ccRCC, KSRP-regulated NEDD4L protein and mRNA expressions were also observed in ACHN pRCC cells (Additional file 1: Fig. S3A, B). In clinical aspects, an inverse correlation between KSRP and NEDD4L was also observed in human ccRCC samples retrieved from TCGA-KIRC (Fig. 2D). As expected, NEDD4L transcripts were significantly lower in primary or metastatic ccRCC tumors compared to normal tissues (Additional file 1: Fig. S4). Moreover, in a normal/tumorous (N/T) paired ccRCC cohort, significantly lower NEDD4L transcripts were also observed in tumors compared to the paired surrounding normal tissues (Fig. 2E). Furthermore, ccRCC patients with lower NEDD4L subsequently had advanced clinical stages (III + IV), more malignant pathological T (T3 + T4) and M (M1) stages (Fig. 2F), and shorter survival times (OS and DSS) (Fig. 2G). Collectively, these findings revealed that NEDD4L may act as a tumor suppressor in ccRCC and participate in KSRP-modulated ccRCC progression.

**Table 2:** Prediction of 29 miRNAs on the 16 potential genes regulated by KH-type splicing regulatory protein (KSRP) via two online available websites

mirRNA/Gene Name	HSPB2	OLFML2B	ASF1A	RHOB	SIPA1L2	NEDD4L	ETV5	LIN7C	ATP2B1	CPEB4	AKT3	ESRRG	PAFAH1B1	GDA	RAB21	CHD9
hsa-miR-4763-3p												H				
hsa-miR-4766-5p											H	H				VH
hsa-miR-1265			VH			H		H	H	H	H		H		VH	VH
hsa-miR-6860																
hsa-miR-5006-5p																
hsa-miR-4483																
hsa-miR-4436a																
hsa-miR-4450																
hsa-miR-6134																
hsa-miR-6721-5p																
hsa-miR-4750-5p																
hsa-miR-6788-5p																
hsa-miR-629-5p					H	H	H				H	H		H		
hsa-miR-4632-5p																
hsa-miR-3909						H							VH			
hsa-miR-8082																
hsa-miR-6809-5p																
hsa-miR-761				H	H	H	H	H	H	VH		H	VH			H
hsa-miR-4419b										H						
hsa-miR-4417									H		H					
hsa-miR-1255b-5p		H				H						H				
hsa-miR-6766-5p																
hsa-miR-4462																
hsa-miR-211-3p																
hsa-miR-6883-5p				H												
hsa-miR-4790-3p																
hsa-miR-6800-5p																
hsa-miR-6781-5p																
hsa-miR-373-5p										H			VH	H	H	H
**MirDIP results**	**0**	**1**	**1**	**2**	**2**	**5**	**2**	**2**	**3**	**4**	**4**	**5**	**4**	**2**	**2**	**4**

VH: Very high (with top 1% prediction score); H: high (with top 5% prediction score)

The bold font represents the total number of miRNA targets predicted to target each gene in the database

**Table 3:** Prediction of 29 miRNAs on the 16 potential genes regulated by KH-type splicing regulatory protein (KSRP) via Stabase online available website

mirRNA/Gene Name	HSPB2	OLFML2B	ASF1A	RHOB	SIPA1L2	NEDD4L	ETV5	LIN7C	ATP2B1	CPEB4	AKT3	ESRRG	PAFAH1B1	GDA	RAB21	CHD9
hsa-miR-4763-3p																
hsa-miR-4766-5p						V		V	V	V	V			V		V
hsa-miR-1265																
hsa-miR-6860																
hsa-miR-5006-5p																
hsa-miR-4483																
hsa-miR-4436a									V				V			
hsa-miR-4450																
hsa-miR-6134																
hsa-miR-6721-5p																
hsa-miR-4750-5p																
hsa-miR-6788-5p																
hsa-miR-629-5p		V		V	V	V	V				V			V		
hsa-miR-4632-5p																
hsa-miR-3909						V							V			
hsa-miR-8082																
hsa-miR-6809-5p																
hsa-miR-761						V	V	V	V	V			V		V	
hsa-miR-4419b																
hsa-miR-4417																
hsa-miR-1255b-5p																
hsa-miR-6766-5p							V	V								
hsa-miR-4462																
hsa-miR-211-3p					V								V			
hsa-miR-6883-5p																
hsa-miR-4790-3p																
hsa-miR-6800-5p																
hsa-miR-6781-5p																
hsa-miR-373-5p			V			V	V	V	V	V	V		V	V	V	V
**Starbase results**	**0**	**1**	**1**	**1**	**2**	**5**	**4**	**4**	**4**	**3**	**3**	**0**	**5**	**3**	**2**	**2**

V: Predicted as a miRNA target.

The bold font represents the total number of miRNA targets predicted to target each gene in the database

### KSRP negatively regulates NEDD4L to trigger EMT-mediated invasion in ccRCC cells

**Fig. 3:**
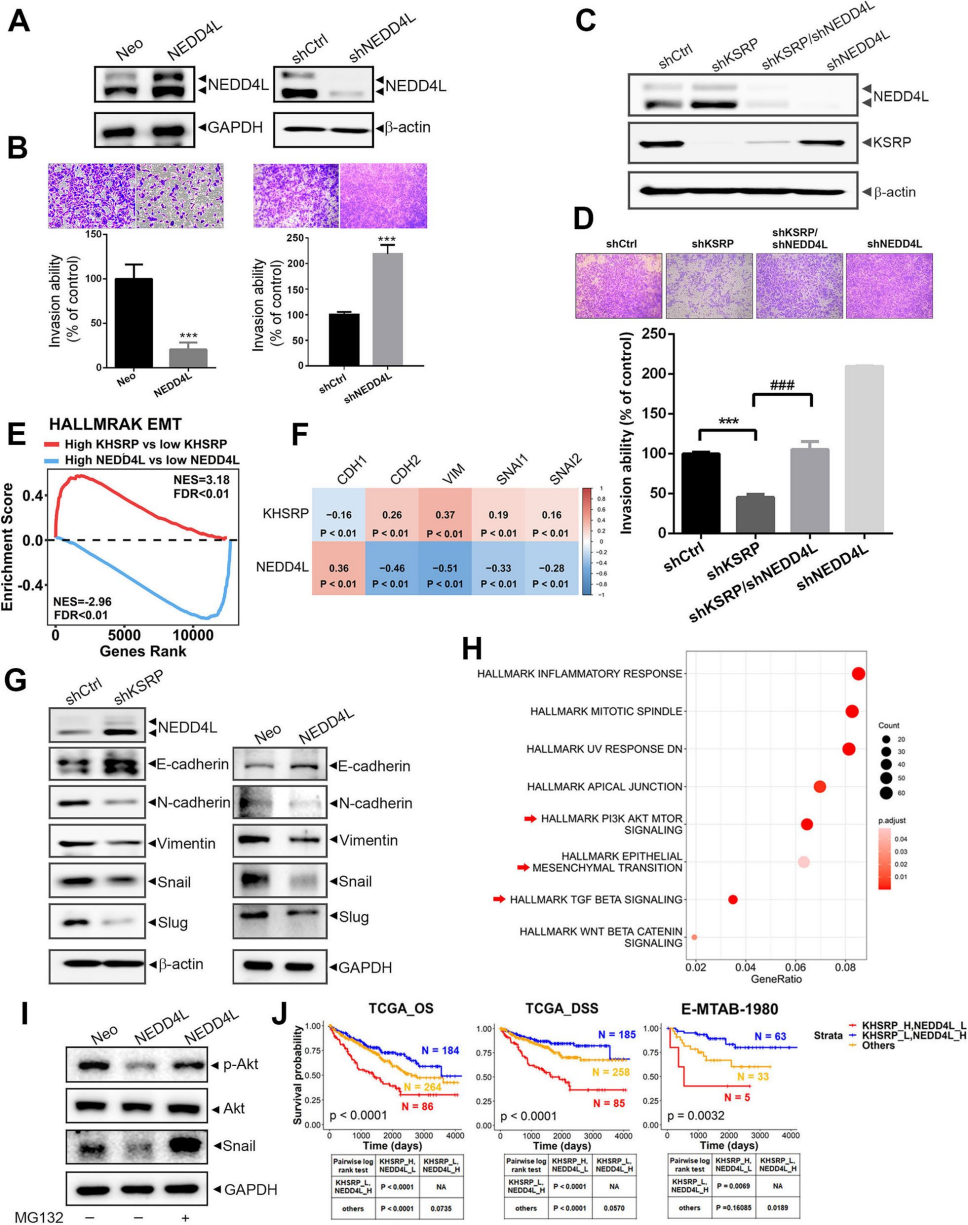
KH-type splicing regulatory protein (KSRP) negatively regulates neural precursor cell-expressed developmentally downregulated 4 like (NEDD4L) to trigger the epithelial-mesenchymal transition (EMT) and promotes invasion of clear cell renal cell carcinoma (ccRCC) cells. **A** NEDD4L was overexpressed (left panel) and knocked-down (right panel) in Caki-1 cells as determined by Western blotting (WB). **B** Invasive abilities of Caki-1 cells overexpressing (left panel) or knocked-down (right panel) NEDD4L were determined by a Matrigel invasion assay. **C, D** Expression levels of NEDD4L and KSRP, and the invasive abilities were respectively determined by WB (**C**) and Matrigel invasion assays (**D**) in Caki-1 cells expressing shKSRP with or without co-expressing shNEDD4L as indicated. Data from **D** are shown as a percent of control cells. Values are presented as the mean ± SD of three independent experiments. ***p < 0.001, compared to the control group. ^###^*p* < 0.001, compared to KSRP shRNA-infected cells. **E** Gene set enrichment analysis of TCGA-KIRC patients with high (top 5%) versus low (bottom 5%) expression of KSRP or NEDD4L. A gene set of the EMT derived from HALLMARK was used. Normalized enrichment scores (NESs) and false discovery rates (FDRs) are shown in the enrichment plot. **F** Correlations of gene expressions of KSRP or NEDD4L with EMT markers are demonstrated in a correlation plot. RNA sequencing data of TCGA-KIRC patients (*n* = 510) were utilized to perform this analysis. Correlation coefficients and *p* values were evaluated by a Pearson correlation analysis. **G** Caki-1 cells expressing either a KSRP shRNA, NEDD4L-expressing, or respective control vector and subjected to WB to determine expressions of EMT-related regulators. **H** Dot plots indicating NEDD4L substrates-enriched signaling. **I** NEDD4L-overexpressing and control Caki-1 cells were treated with or without 20 μM MG-132 for 4 h, and then the Akt, p-Akt, Snail, and GAPDH proteins were analyzed by WB. **J** All ccRCC patients from TCGA or E-MTAB-1980 were separated into a negative correlation with KSRP and NEDD4L expressions (low KSRP with high NEDD4L and high KSRP with low NEDD4L) and others. Data showed that patients in the KSRP^high^/NEDD4L^low^ group had the worst prognosis

To further ascertain the role of NEDD4L in KSRP-modulated ccRCC progression, we first examined whether NEDD4L regulates cell invasion in ccRCC cells. We conducted NEDD4L overexpression and knockdown in ccRCC cells using an NEDD4L-expressing vector and NEDD4L-specific shRNA, respectively (Fig. 3A; Additional file 1: Fig. S5A). NEDD4L overexpression or depletion respectively resulted in a significant decrease or increase in the invasive ability of ccRCC cells (Fig. 3B; Additional file 1: Fig. S5B), suggesting that NEDD4L suppresses ccRCC cell invasion. In addition, we further observed that upregulation of NEDD4L and inhibition of invasion caused by KSRP-KD could be significantly reversed by co-depletion of NEDD4L in both ccRCC (Caki-1 and 786-O) and pRCC (ACHN) cells (Fig. 3C, D; Additional file 1: Fig. S6A, B), suggesting that suppression of NEDD4L is critical for KSRP-induced invasiveness of renal cell carcinoma cells. To further decipher the mechanism underlying KSRP-NEDD4L axis-modulated ccRCC progression, GSEA and Pearson correlation analyses based on TCGA-KIRC dataset were performed. We identified that EMT-associated gene signatures were enriched in ccRCC with high KSRP expression, while high NEDD4L expression was inversely related to the EMT (Fig. 3E). We then verified correlations of KSRP or NEDD4L with EMT-related genes in human ccRCC samples using the cBioPortal platform and observed that KSRP and NEDD4L expressions were respectively positively and negatively correlated with expressions of the mesenchymal markers of N-cadherin (CDH2), vimentin (VIM), Snail (SNAI1), and Slug (SNAI2) (Fig. 3F). Consistently, downregulation of mesenchymal markers and upregulation of an epithelial marker, E-cadherin (CHD1), were observed in Caki-1 and 786-O ccRCC cells with KSRP-KD or NEDD4L overexpression (Fig. 3G; Additional file 1: Fig. S7). To further identify potential targets of the NEDD4L ubiquitin ligase, we used ubiBrowser to search for potential substrates. As shown in Additional file 1: Figs. S8, 57 known and 2378 predicted substrates were found. Combining the known and predicted substrates in a pathway enrichment analysis showed that NEDD4L’s substrates were involved in EMT-related pathways or upstream pathways of the EMT such as phosphatidylinositol 3-kinase (PI3K)/Akt/mammalian target of rapamycin (mTOR) and transforming growth factor (TGF)-β signaling [21] (Fig. 3H). Next, we found that pretreatment of Caki-1 and 786-O cells with the proteasome inhibitor, MG-132, could prevent decreases in p-Akt and Snail levels after overexpressing NEDD4L (Fig. 3I; Additional file 1: Fig. S9). Taken together, these results indicated that KSRP negatively regulated NEDD4L expression to prevent degradation of EMT upstream signals and subsequently induced an EMT-mediated invasive ability in ccRCC cells. The same aforementioned TCGA-KIRC and E-MTAB-1980 datasets all indicated that ccRCC patients with KSRP^high^/NEDD4L^low^ had the worst prognoses (OS or DSS) compared to those with KSRP^low^/NEDD4L^high^ or other expression statuses (Fig. 3J), and these clinical data further confirmed that upregulation of KSRP and downregulation of NEDD4L are critical events in promoting ccRCC progression.

### Negative regulation of NEDD4L by KSRP promotes progression of ccRCC in vivo

**Fig. 4:**
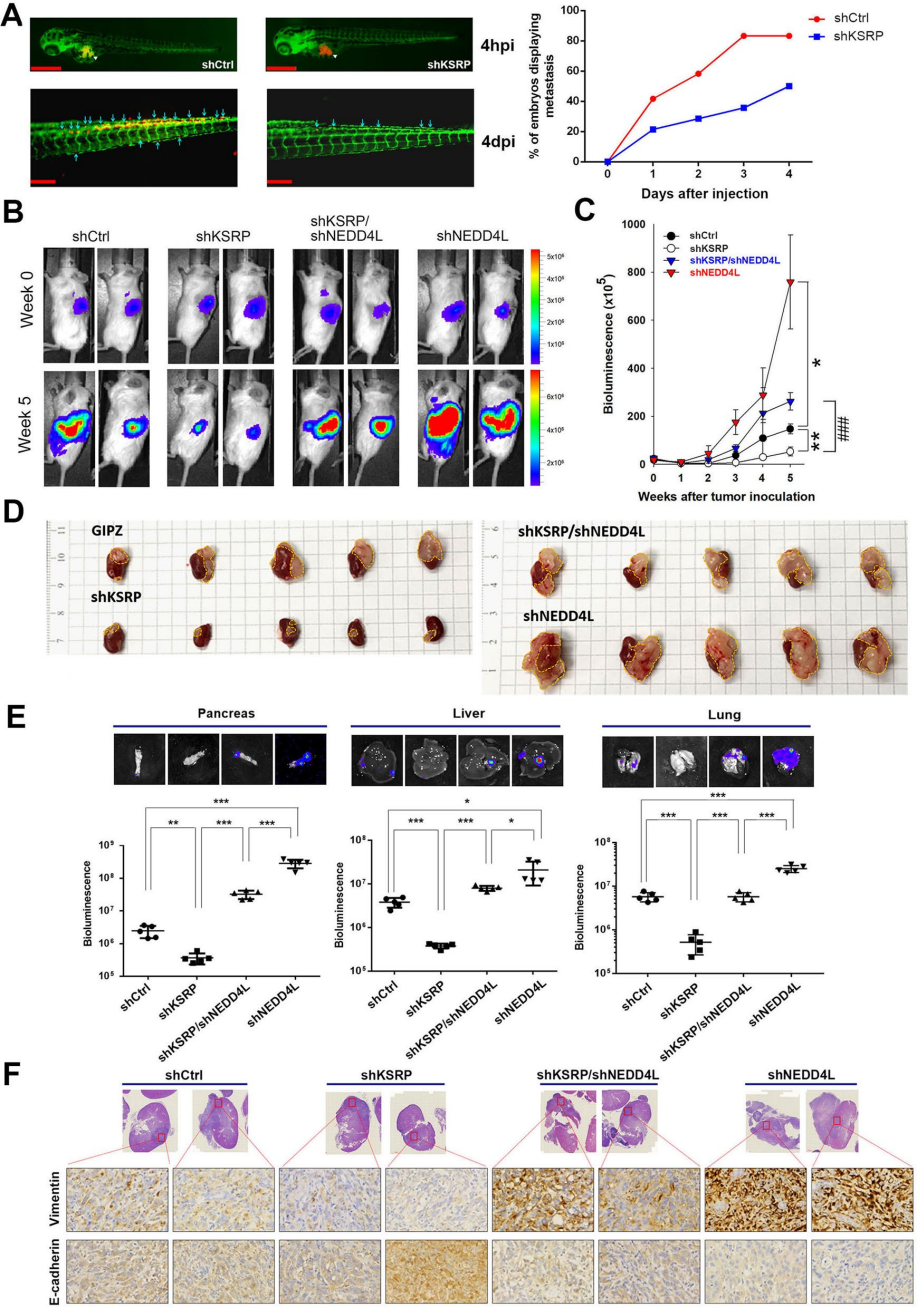
Oncogenic role of KH-type splicing regulatory protein (KSRP) in vivo. **A** Dissemination of human clear cell renal cell carcinoma (ccRCC) cells in zebrafish embryos. Caki-1 cells were implanted into 48 h post-fertilization zebrafish embryos. Tumor cell dissemination was detected at day 4 post-injection. White arrows indicate the primary tumor and blue arrowheads indicate disseminated tumor foci. Frequencies of fish showing trunk and end-tail dissemination were monitored and counted every day (right panel). shCtrl: n = 12; shKSRP: n = 14. 4hpi, 4 h post-injection; 4dpi, 4 days post-injection. **B** Male NOD/SCID mice were orthotopically injected with luciferase-tagged and KSRP-knockdown (shKSRP), neural precursor cell-expressed developmentally downregulated 4 like (NEDD4L)-knockdown (shNEDD4L), or double-knockdown (shKSRP/shNEDD4L) Caki-1 cells. Whole-body bioluminescence imaging was conducted at the indicated time points after injecting cells into mice. **C** Quantitative analysis of Xenogen imaging signal intensity (photons/s/cm^2^/sr) every week. *p < 0.05, **p < 0.01, compared to the control group. ^###^*p* < 0.001 compared to the KSRP-knockdown only group. **D** Gross appearance of orthotopic tumors (indicated by the yellow dashed line) in a kidney. **E** Upper panel, Representative ex vivo bioluminescence imaging of metastatic sites (pancreas, liver, and lungs) at the end of this in vivo study. Lower panel, Signal intensities of metastatic organs were imaged with bioluminescence at the end of the study, with the mean signal for each group indicated. *p < 0.05, **p < 0.01, ***p < 0.001. **F** Caki-1 xenografts infected with the indicated shRNAs were isolated to detect expressions of vimentin and E-cadherin by IHC staining. Original magnification, 400 ×

We next investigated the in vivo effects of the KSRP-NEDD4L axis on ccRCC progression. First, we established a zebrafish xenograft metastasis model by transplanting DiI dye-labeled Caki-1 and 786-O cells expressing shKSRP or shCtrl in transgenic *Tg(fli1: EGFP)* zebrafish embryos which led to extensive dissemination of tumor cells. At 24 h post-injection, frequencies of fish showing metastatic dissemination of inoculated cells were measured every day using fluorescence microscopy. In fish that were inoculated with KSRP-KD Caki-1 cells, the frequencies of fish showing trunk and end-tail dissemination dramatically decreased compared to fish that were inoculated with control cells (Fig. 4A). Moreover, lower embryonic death was also observed in fish that were inoculated with KSRP-KD 786-O cells compared to the control group (Additional file 1: Fig. S10). We further examined the effect of the KSRP-NEDD4L axis in regulating ccRCC progression in a ccRCC-bearing mouse model through orthotopically injecting luciferase-expressing Caki-1 cells (Caki-1-shCtrl-luciferase, Caki-1-shKSRP-luciferase, Caki-1-shNEDD4L-luciferase, or Caki-1-shKSRP/shNEDD4L-luciferase) into NOD-SCID mice. We observed that after 5 weeks, SCID mice implanted with control cells (Caki-1/shCtrl) respectively produced larger and smaller tumors than did Caki-1/shKSRP and Caki-1/shNEDD4L cells injected into mice, as revealed by photon emission detection (Fig. 4B, C). After the mice were sacrificed at the end of the experiment, visible tumor masses from tumor-implanted kidneys also showed similar trends with the tumor size detected by in vivo images (Fig. 4D). Moreover, ex vivo imaging of the excised pancreas, liver, and lungs showed significantly lower photon intensities in Caki-1/shKSRP-injected mice compared to control mice, and these results were consistent with observations in zebrafish metastasis models. In contrast, a metastasis-promoting effect was observed in mice injected with Caki-1/shNEDD4L cells (Fig. 4E). To further examine the effects of the KSRP-NEDD4L axis on the EMT in vivo, an IHC analysis was used to determine the EMT-related markers. Consistent with the in vitro findings, we observed that E-cadherin and N-cadherin expression levels were respectively increased and decreased in tumor tissues from Caki-1/shKSRP-injected mice and the opposite results were observed in tissues from Caki-1/shNEDD4L-injected mice (Fig. 4F). As expected, all of these phenomena observed in KSRP-depleted cells could also be significantly reversed when NEDD4L was knocked-down (Fig. 4B–F), suggesting that KSRP exerts negative regulation on NEDD4L expression to promote the EMT and subsequent ccRCC progression in vivo.

### KSRP modulates mRNA stability of NEDD4L through upregulating miR-629-5p and targeting ARE sites of the 3′UTR

**Fig. 5:**
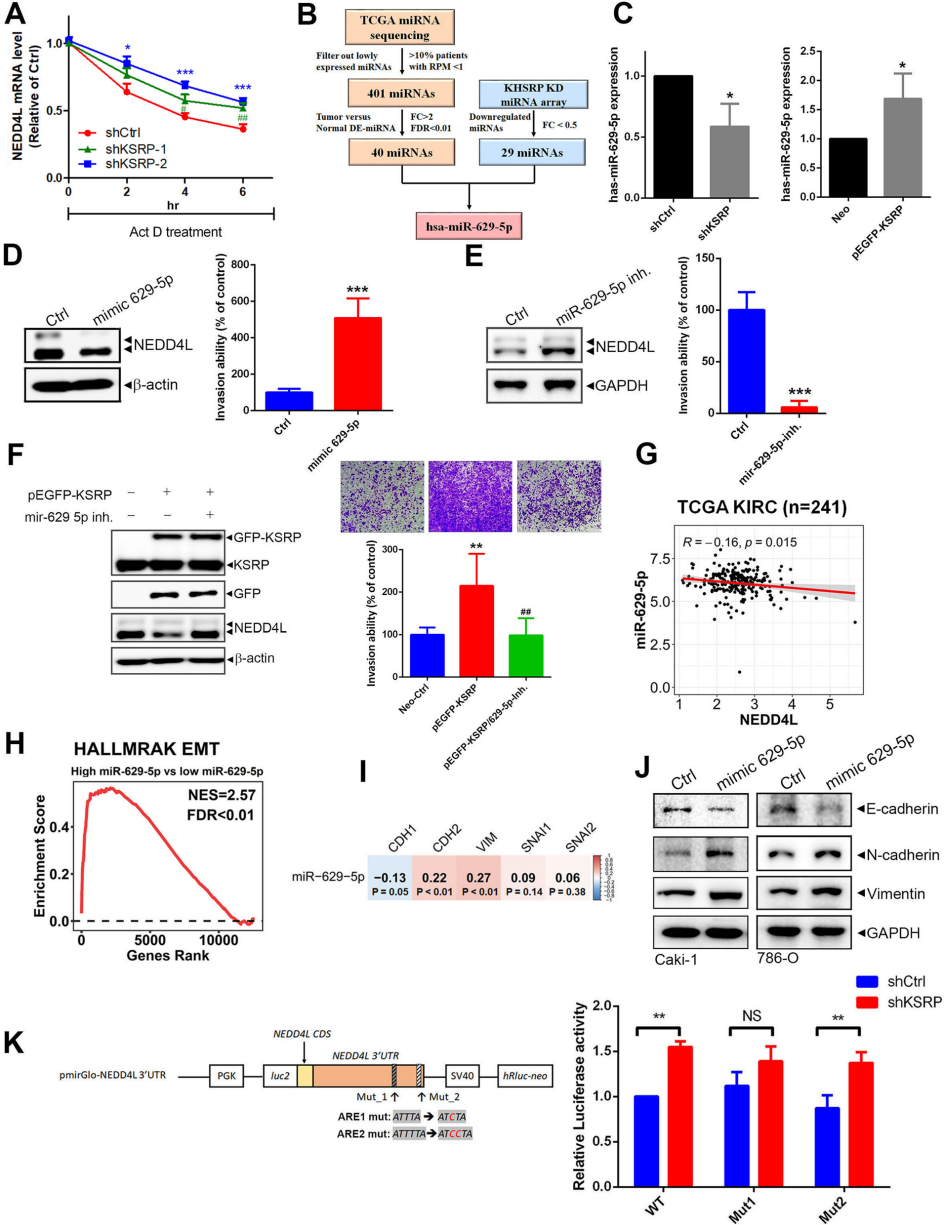
KH-type splicing regulatory protein (KSRP) negatively regulates neural precursor cell-expressed developmentally downregulated 4 like (NEDD4L) mRNA stability via inducing miR-629-5p upregulation and targeting AU-rich elements (AREs) of the 3' untranslated region (3’UTR). **A** Decay rate of NEDD4L mRNA after treatment with 5 μg/mL actinomycin D for the indicated times in Caki-1 cells with or without KSRP-knockdown. **B** Flowchart for identifying miRNA induced by KSRP. The left part shows that TCGA miRNA sequencing data were retrieved from UCSC Xena. TCGA KIRC patients with low expression of miRNA (> 10% patients with reads per million mapped reads (RPM) of < 1) were excluded. The remaining 401 miRNAs were used to perform a differentially expressed (DE)-miRNA analysis between tumor (*n* = 241) and normal tissues (*n* = 70). Forty miRNAs were identified as upregulated miRNAs (with fold change (FC) of > 2 and a false discovery rate (FDR) of < 0.01) in KIRC tumor tissues. Right part shows 29 miRNAs with an FC of < 0.5 as identified in KSRP-depleted Caki-1 cells. By intersecting miRNAs upregulated in TCGA KIRC tissues with miRNAs induced by KSRP in ccRCC cells, miR-629-5p was identified as a critical candidate. **C** Real-time qPCR analysis of miR-629-5p in Caki-1 cells transfected with a KSRP shRNA, pEGFP-KSRP, or their corresponding control vector. **D, E** Caki-1 cells were transfected with an miR-629-5p mimic (**D**) or miR-629-5p inhibitor (**E**) for 24 h. NEDD4L protein levels and invasive ability of cells were respectively determined by Western blot (WB) (left panel) and Matrigel invasion (right panel) assays. **F** Expressions of KSRP and NEDD4L (left panel) and invasive ability of cells (right panel) were determined in Caki-1 cells transfected with pEGFP-KSRP with or without an miR-629-5p inhibitor. **G** Correlation between miR-629-5p and NEDD4L expression in TCGA KIRC patients as demonstrated in dot plots. Correlation coefficients and *p* values were evaluated by a Pearson correlation analysis. **H** GSEA of TCGA KIRC patients with high (top 5%) versus low (bottom 5%) expressions of miR-629-5p. A gene set of the epithelial-mesenchymal transition (EMT) derived from HALLMARK was used. The normalized enrichment score (NES) and FDR are shown in the enrichment plot. **I** Correlations of miR-629-5p expression with EMT markers were demonstrated in a correlation plot using miRNA sequencing data of TCGA KIRC patients. Correlation coefficients and *p* values were evaluated by a Pearson correlation analysis. **J** WB analysis of E-cadherin, N-cadherin, and vimentin expressions in Caki-1 and 786-O cells transfected with an miR-629-5p mimic. **K** Left panel, Schematic of the human NEDD4L-3' untranslated region (3'UTR) inserted in the luciferase reporter vector. Positions of AU-rich elements (AREs) are indicated by two gray oblongs, and the corresponding sequences are shown underneath. Right panel, Relative luciferase activities of Caki-1 cells transfected with the NEDD4L luciferase 3’UTR reporter vector containing wild-type or mutant ARE domains with or without KSRP shRNA. Values are presented as the mean ± SD of three independent experiments. **p < 0.01, compared to the respective control groups

Since KSRP was shown to control mRNA turnover at various levels including promotion of miRNA biogenesis or direct targeting of ARE sites on the 3′UTR of mRNA [6], we first investigated whether KSRP regulates NEDD4L expression through decreasing its mRNA stability. Transcription of nascent mRNA was prevented by actinomycin D treatment in KSRP-KD cells, and the remaining mRNA was monitored by an RT-qPCR to determine the RNA half-life. As shown in Fig. 5A and Additional file 1: Fig. S11, the NEDD4L mRNA half-life in Caki-1 and 786-O was longer in shKSRP cells, including two independent shRNA clones, compared to that in shCtrl cells. From results of the miRNA microarray, we identified 29 downregulated miRNAs in KSRP-KD Caki-1 cells (Additional file 1: Fig. S12A). Since KSRP was elevated and exhibited an oncogenic role in ccRCC, we next identified 40 upregulated miRNA candidates in ccRCC tumor tissues compared to normal tissues using TCGA miRNA sequencing data (Additional file 1: Fig. S12B). By intersecting miRNAs upregulated in TCGA KIRC tissues with downregulated miRNAs caused by KSRP depletion in ccRCC cells, miR-629-5p is the sole intersection. The detailed process of miRNA selection is shown in Fig. 5B. Actually, results of the RT-qPCR showed that significant downregulation and upregulation of mature miR-629-5p level were respectively observed in KSRP-KD and -overexpressing Caki-1 and 786-O cells (Fig. 5C; Additional file 1: Fig. S13A). Additionally, the level of primary miR-629 (pri-miR-629) was reduced upon KSRP overexpression in ccRCC cells (Additional file 1: Fig. S13B). This finding suggests that KSRP may play a role in facilitating the biogenesis of miR-629-5p. Data in Table 2 and 3 show that NEDD4L is one of the predicted targets of miR-629-5p. We then transfected miRNA mimic or an inhibitor of miR-629-5p into Caki-1 and 786-O cells and found that expression of the miR-629-5p mimic resulted in decreased NEDD4L expression (Fig. 5D, left panel; Additional file 1: Fig. S14A, up panel) and increased cell invasion (Fig. 5D, right panel; Additional file 1: Fig. S14A, down panel), while expression of the miR-629-5p inhibitor caused opposite effects (Fig. 5E; Additional file 1: Fig. S14B). Furthermore, KSRP-overexpression-induced downregulation of NEDD4L mRNA (Additional file 1: Fig. S15A) and protein levels and upregulation of the invasive ability were all significantly reversed in the presence of an miR-629-5p inhibitor in ccRCC cells (Fig. 5F; Additional file 1: Fig. S15B). In the clinic, there was an elevation in miR-629-5p expression observed in paired cancerous tissues compared to adjacent normal tissues (Additional file 1: Fig. S16A). In addition, we found that miR-629-5p expression levels were significantly higher in primary and metastatic ccRCC tumors compared to normal tissues (Additional file 1: Fig. S16B), were inversely correlated with NEDD4L levels (Fig. 5G), and were associated with poor OS and DSS in patients with ccRCC (Additional file 1: Fig. S17). A GSEA analysis showed that high expression of miR-629-5p was associated with EMT activation (Fig. 5H). A Pearson correlation analysis also showed that miR-629-5p expression was positively associated with expressions of mesenchymal markers (CDH2 and VIM), but it was negatively correlated with an epithelial marker, CDH1 (Fig. 5I). Furthermore, upregulation of mesenchymal markers (N-cadherin and vimentin) and downregulation of epithelial marker E-cadherin was observed in ccRCC cells which overexpressed the miR-629-5p mimic (Fig. 5J). Taken together, these results indicated that miR-629-5p may play a critical role in KSRP-regulated NEDD4L mRNA stability and subsequent EMT-induced cancer progression. In addition to miRNA biogenesis, the ARE is another common determinant of RNA stability regulated by KSRP [6]. By analyzing NEDD4L mRNA with online ARE analytical software (AREsite2), two ARE sites in the 3′UTR were identified. We next mutated each ARE site as ARE1 mut and ARE2 mut to further evaluate the importance of these two ARE sites (Fig. 5K, left panel; Additional file 1: Fig. S18). Similar to the non-mutated 3’UTR reporter, we observed that activity of ARE2 mut reporter still increased by KSRP-KD, while the activity of the ARE1 mut reporter did not significantly increase (Fig. 5K, right panel; Additional file 1: Fig. S18). These results suggested that the ARE1 site of the NEDD4L 3’UTR is critical for KSRP binding to regulate NEDD4L mRNA stability.

### KSRP exhibits a transcriptionally suppressive effect on NEDD4L through induction of the transcriptional repressor, WT1

**Fig. 6:**
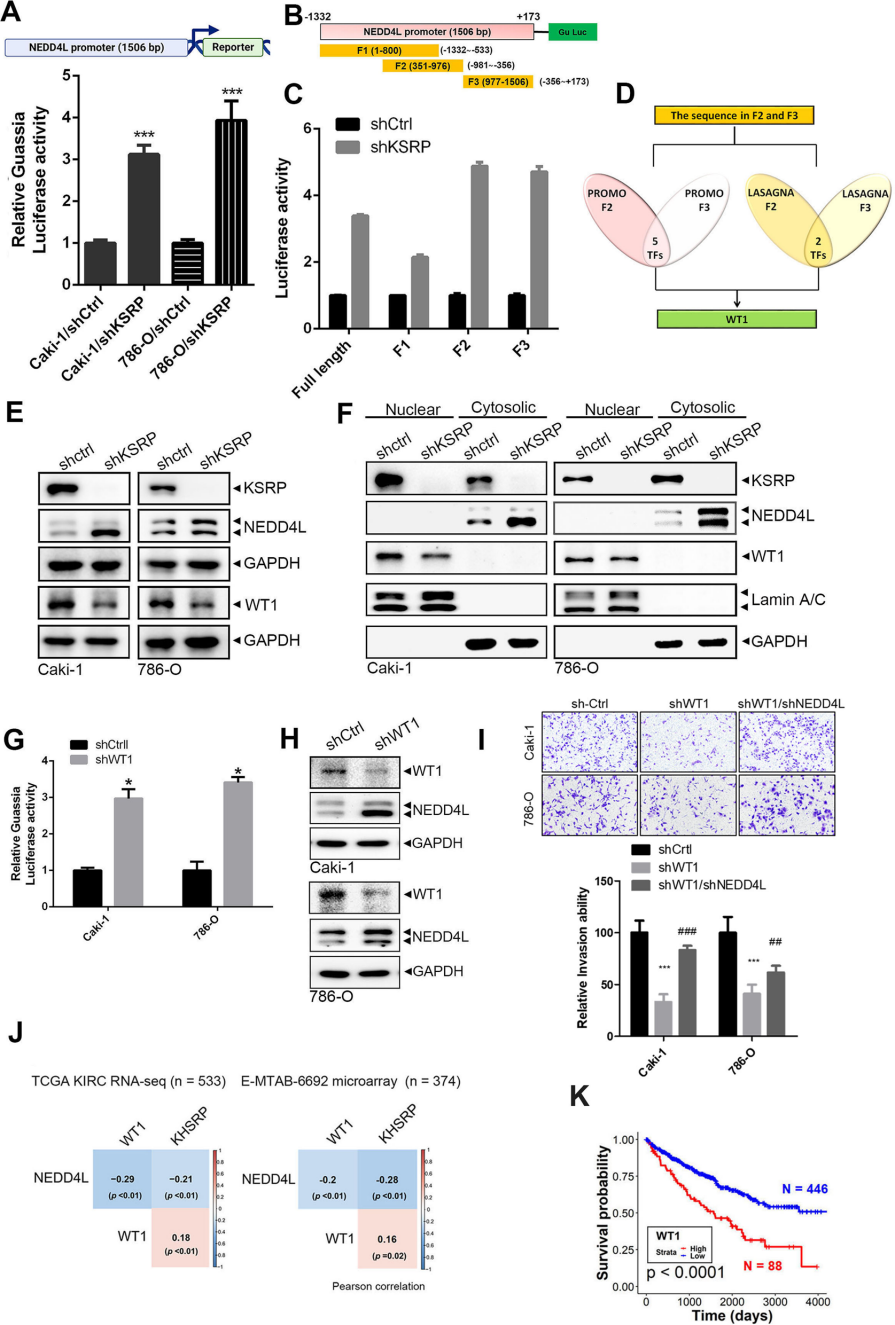
KH-type splicing regulatory protein (KSRP) transcriptionally suppresses neural precursor cell-expressed developmentally downregulated 4 like (NEDD4L) via inducing upregulation of Wilm's tumor 1 (WT1). **A** Upper panel, schematic representation of the promoter region (1506 bp) of NEDD4L. Lower panel, relative luciferase activities of Caki-1 cells transfected with the NADD4L promoter reporter with or without KSRP shRNA. *** *p* < 0.001, compared to the control group. **B** Schematic illustration of truncated NEDD4L promoter constructs (F1–F3). The position of the yellow box represents the fragment location relative to the full-length NEDD4L. **C** Luciferase reporter activity driven by different NEDD4L promoter segments in Caki-1 cells infected with shKSRP or shCtrl. **D** Flowchart for identifying possible transcription factors (TFs) involved in KSRP-regulated NEDD4L promoter activity. TF binding to F2 or F3 fragments was predicted by the PROMO and LASAGNA websites. Common TF-binding regions on F2 and F3 were collected, and WT1 was ultimately identified as a candidate TF. **E–F** WB analysis of KSRP, WT1, and NEDD4L expression levels in whole-cell lysates (**E**) or cytoplasmic and nuclear fractions (**F**) from Caki-1 and 786-O cells infected with shKSRP or shCtrl. **G-H** Luciferase reporter (**G**) and WB (**H**) assays were respectively used to detect promoter activities and expression levels of NEDD4L in Caki-1 and 786-O cells infected with shWT1 or shCtrl. **I** Caki-1 and 786-O cells were infected with shCtrl, shWT1 or shWT1 + shNEDD4L, and the invasive ability of cells were respectively determined by Matrigel invasion assays. **J** Correlations among KSRP, NEDD4L, and WT1 were analyzed in clear cell renal cell carcinoma (ccRCC) patients from TCGA KIRC and E-MTAB-6692 datasets. Correlation coefficients and *p* values were evaluated by a Pearson correlation analysis. **K** Kaplan–Meier analysis of overall survival (OS) rates in patients with ccRCC presenting with high or low expression of WT1 using data from TCGA KIRC dataset

In addition to the post-transcriptional regulation of NEDD4L by KSRP, a previous study also showed that KSRP could transcriptionally regulate its target genes, such as c-Myc in nuclei [6]. Herein, we observed that KSRP-KD significantly increased NEDD4L promoter activity in Caki-1 and 786-O cells (Fig. 6A). To explore the detailed region that is responsible for KSRP-regulated promoter activity of NEDD4L, a series of NEDD4L gene promoters with sequential deletions was constructed as F1 (− 1332 to − 533), F2 (− 981 to − 356), and F3 (− 356 to  + 173) (Fig. 6B). The promoter region of F1, but not F2 or F3, resulted in decreased transcriptional activity compared to the full-length promoter in KSRP-KD cells (Fig. 6C; Additional file 1: Fig. S19), suggesting the F2 and F3 promoter regions of NEDD4L are critical for KSRP-regulated promoter activity. We further analyzed putative transcription factor (TF)-binding sites in the F2 and F3 regions of the NEDD4L promoter by PROMO [22] and LASAGNA [23] algorithms which predicted that binding sites of WT1 were located in the F2 and F3 regions of the NEDD4L promoter (Fig. 6D). In KSRP-depleted Caki-1 and 786-O cells, we observed the downregulation of WT1 compared to control cells (Fig. 6E), while overexpression of KSRP induced upregulation of this TF in both mRNA and protein level (Additional file 1: Fig. S20). To further detect the subcellular localization of KSRP, NEDD4L, and WT1 in ccRCC cells, a Western blot analysis was performed after separation of the cytoplasm and nuclei of Caki-1 and 786-O cells. Figure 6F shows that KSRP expression was detected in both cellular fractions, while WT1 and NEDD4L were respectively detected mainly in nuclei and the cytoplasm. KSRP-KD also induced downregulation of WT1 in nuclei and upregulation of NEDD4L in the cytoplasm. WT1 was reported to be a dichotomous TF that can either activate or repress transcription of target genes [24]. Herein, lentiviral-based shRNA was used to directly knock-down WT1 in Caki-1 and 786-O cells, and we observed that depletion of WT1 induced an increase in NEDD4L promoter activity (Fig. 6G) and protein expression (Fig. 6H) in both cells, suggesting that WT1 might act as a transcriptional repressor induced by KSRP to transcriptionally suppress NEDD4L in ccRCC cells. Functionally, we also observed the invasion ability was significantly decreased caused by WT1 knockdown and could be reversed by co-depletion of NEDD4L in both Caki-1 and 786-O cells (Fig. 6I). That suggested WT1 has a pro-oncogenic function in ccRCC via regulating NEDD4L. Consistent with our in vitro results, expression levels of WT1 were positively correlated with KSRP and negatively correlated with NEDD4L levels in tumor specimens from two independent ccRCC cohorts (Fig. 6J). Moreover, ccRCC patients with higher WT1 expression levels had shorter survival times (Fig. 6K). Collectively, these findings revealed that WT1 may act as an oncogene in ccRCC and participate in KSRP-induced transcriptional suppression of NEDD4L.

## Discussion

Recently, accumulating evidence indicates that the RBP, KSRP, is involved in modulating tumor progression, but the consequences of altered KSRP expression in different tumor types are conflicting. KSRP seems to be a multi-functional protein with different capacities for executing its effects on target mRNAs [6]. In the present study, we found that KSRP was upregulated in ccRCC tissues and was associated with poor clinical outcomes. Depletion of KSRP suppressed the proliferation and metastasis of ccRCC cells both in vitro and in vivo. Further molecular studies revealed that KSRP promotes progression of ccRCC via inducing NEDD4L mRNA destabilization and transcriptional inhibition, and subsequently triggering the EMT. Upregulation of miR-629-5p and ARE binding of the NEDD4L 3′UTR were involved in KSRP-mediated decay of NEDD4L mRNA. Increasing expression of the WT1 transcriptional repressor participated in KSRP-mediated transcriptional inhibition of NEDD4L. To our best knowledge, this is the first report identifying NEDD4L as a novel target of KSRP for promoting ccRCC progression. In this study, the correlation among the transcripts of KSRP, WT1, NEDD4L, and miR-629-5p in ccRCC tissues was determined by online databases. However, it is important to note that a limitation of our research is the absence of our own ccRCC specimens to validate the correlation among the protein levels of KSRP, WT1, and NEDD4L and the transcript level of miR-629-5p. We recognize the significance of addressing this limitation in future research to further investigate the relationships among these molecules in ccRCC.

Conflicting roles of KSRP during cancer development may be due to the various genes targeted by KSRP and may highly depend on the cancer type and cell content [6]. In the present study by employing multiple in vitro and in vivo assays combined with clinical analyses, KSRP was ascertained to be an oncoprotein in ccRCC. Next, cDNA and miRNA arrays were utilized to identify NEDD4L as a potential target gene negatively regulated by KSRP in ccRCC cells. NEDD4L is an E3 ubiquitin ligase and was reported to function as a tumor suppressor in most cancer types via elevating the degradation of diverse substrates [20]. For example, NEDD4L can degrade serine/threonine kinase 35 (STK35) by ubiquitination and subsequently inhibit glycolysis and induce apoptosis of colorectal cancer cells via inhibiting the Akt signaling pathway [25]. NEDD4L was shown to suppress tumorigenesis of breast cancer by degrading copper transporter 1 (CTR1) and subsequently inhibiting Akt signaling [26]. In gliomas, ubiquitination of sphingosine kinase 2 (SphK2) by NEDD4L was shown to suppress invasion of cells via blocking the AKT/β-catenin pathway [27]. In our study, knockdown and overexpression of NEDD4L respectively promoted and suppressed the invasive ability of ccRCC cells, and NEDD4L-KD significantly rescued the invasion and metastasis suppression imposed by KSRP-KD in Caki-1 cells and the Caki-1 xenograft model. These results all suggest that NEDD4L also acts as a tumor suppressor in ccRCC as well as a critical downstream effector of KSRP-induced ccRCC metastasis. Through the prediction and analysis of putative substrates ubiquitinated by NEDD4L, we found that NEDD4L’s putative substrates were enriched in an EMT-related pathway or upstream regulatory pathways of the EMT such as PI3/AKT/mTOR and TGF-β signaling [28]. Indeed, we observed that both KSRP-KD and NEDD4L overexpression suppressed expression of EMT-related markers in ccRCC cells. Pretreatment with the proteasome inhibitor, MG-132, was able to prevent decreases in p-Akt and Snail after NEDD4L overexpression, while total Akt was not affected by NEDD4L. Thus, we proposed that NEDD4L can inhibit Akt activation in ccRCC possibly through degrading its upstream activators such as STK35, SphK2, CTR1, and PIK3CA, which were previously reported in different cancers [25–27, 29]. Whether these potential substrates of NEDD4L participate in NEDD4L-regulated Akt activation in ccRCC cells needs to be investigated in the future. In ccRCC clinical samples, we further observed that KSRP and NEDD4L expression levels were respectively positively and negatively correlated with EMT-associated gene signatures. Collectively, these results suggest that KSRP negatively regulates NEDD4L expression to promote ccRCC progression via triggering the Akt-mediated EMT.

Mechanistic investigations from an miRNA array combined with several miRNA target prediction programs and TCGA-KIRC miRNA sequencing data showed that miR-629-5p expression was upregulated in ccRCC and might be induced by KSRP to target NEDD4L. Mir-629-5p was reported to be an oncogene which promotes the progression of several cancers such as prostate and liver cancers via targeting different genes [30, 31]. Herein, we demonstrated that miR-629-5p played a critical role in the KSRP-mediated decrease of NEDD4L mRNA stability and increase of EMT-mediated invasion in ccRCC cells. KSRP was indicated as being a component of both Drosha and Dicer complexes and promoting the biogenesis of a subset of miRNAs from pri-miRNA to mature miRNA [9]. Moreover, a study by Yuan et al. reported that sumoylation of KSRP reduces its binding to the pri-miRNA/Drosha complex, particularly in the case of pri-miRNAs containing G-rich stretches on their terminal loops (TL-G-rich miRNAs) [32]. Yuan et al. indicated that miR-629-5p is likely to belong to the TL-G-rich miRNAs, and its biogenesis may be influenced by the sumoylation status of KSRP in the prostate cancer cell line DU145. Herein, we did observe the decrease of pri-miR-629-5p (Additional file 1: Fig. S13B) and increase of mature miR-629-5p (Fig. 5C; Additional file 1: Fig. S13A) in ccRCC cells overexpressing KSRP, suggesting KSRP can promote the biogenesis of matured miR-629-5p in ccRCC. Thus, we suggest that KSRP promote maturation of miR-629-5p to induce NEDD4L mRNA destabilization in ccRCC cells. However, the sumoylation status of KSRP and the impact of sumoylated KSRP on the biogenesis of miR-629-5p in ccRCC cells need to be further investigated in our future work. In addition to miR-629-5p, miR-23a was shown to promote the progression of pancreatic cancer via targeting NEDD4L [33]. Our previous study showed that KSRP can promote the maturation of miR-23a to regulate lung cancer progression [12]. In our screening (Tables 2  and 3), although miR-23a was not identified as one of the putative miRNAs regulated by KSRP in ccRCC cells, it is still possible that miR-23a is involved in KSRP-mediated NEDD4L mRNA decay in ccRCC, but this issue needs to be further determined.

Many genes that are involved in cancer progression possess AREs, which are *cis*-acting mRNA decay determinants, located in the 3’UTR, and targeted by RBPs [34]. For example, the RBP, tristetraprolin (TTP), was reported to promote mRNA decay of the EMT-related markers, Snail and Twist1, in an ARE-dependent manner [35]. Similar to TTP, KSRP was also shown to interact with and destabilize mRNAs containing AREs in their 3’UTR [8]. Herein, we found two ARE sites in the 3’UTR of NEDD4L, and the NEDD4L 3’UTR reporter activity regulated by KSRP was abolished when the first ARE site was mutated, suggesting that KSRP may bind to a specific ARE in the NEDD4L 3′UTR to regulate its mRNA stability. Similar to our result, another RBP, AU-binding factor 1 (AUF1), was shown to degrade NEDD4L by interacting with AREs of NEDD4L in renal proximal tubule cells [36]. KSRP and AUF1 were reported to show a cooperative mode involved in Pin1-modulated mRNA stability [37]. The crosstalk of KSRP and AUF1 in regulating NEDD4L decay is worthy of future investigations.

In addition to the post-transcriptional regulation of NEDD4L by KSRP in the cytosol of ccRCC cells, KSRP was also reported to directly regulate transcription by interacting with the promoter, or it might possibly control a factor involved in gene transcription in nuclei [6, 8]. Herein, we found that manipulation of KSRP significantly affected the promoter activity and mRNA level of NEDD4L in ccRCC cells, suggesting the transcriptional regulation of the NEDD4L gene by KSRP. Moreover, we identified WT1 as a possible important transcriptional regulator which can be induced by KSRP and suppress NEDD4L promoter activity, implying that WT1 is involved in KSRP-modulated NEDD4L promoter activity in ccRCC cells. WT1 was reported to manifest both tumor suppressive and oncogenic activities in different cancer types due to its interaction with various proteins leading to transcriptional activation or repression of different genes. For example, prostate apoptosis response (Par)-4 was reported to interact with WT1 in nuclei causing transcriptional repression of WT1’s target genes [24, 38]. DNA-binding factors such as p53 were shown to interact with WT1 and switch WT1 from a transcriptional activator to a repressor [39]. p73 was also reported to bind to WT1 and inhibit its transcriptional activity and DNA-binding ability [40]. Our study showed that KSRP exhibited transcriptional inhibition of the NEDD4L gene via inducing WT1 upregulation. Whether KRSP interacts with WT1 in nuclei to regulate NEDD4L promoter activity in ccRCC cells remains to be further clarified in future work.

## Conclusions

**Fig. 7:**
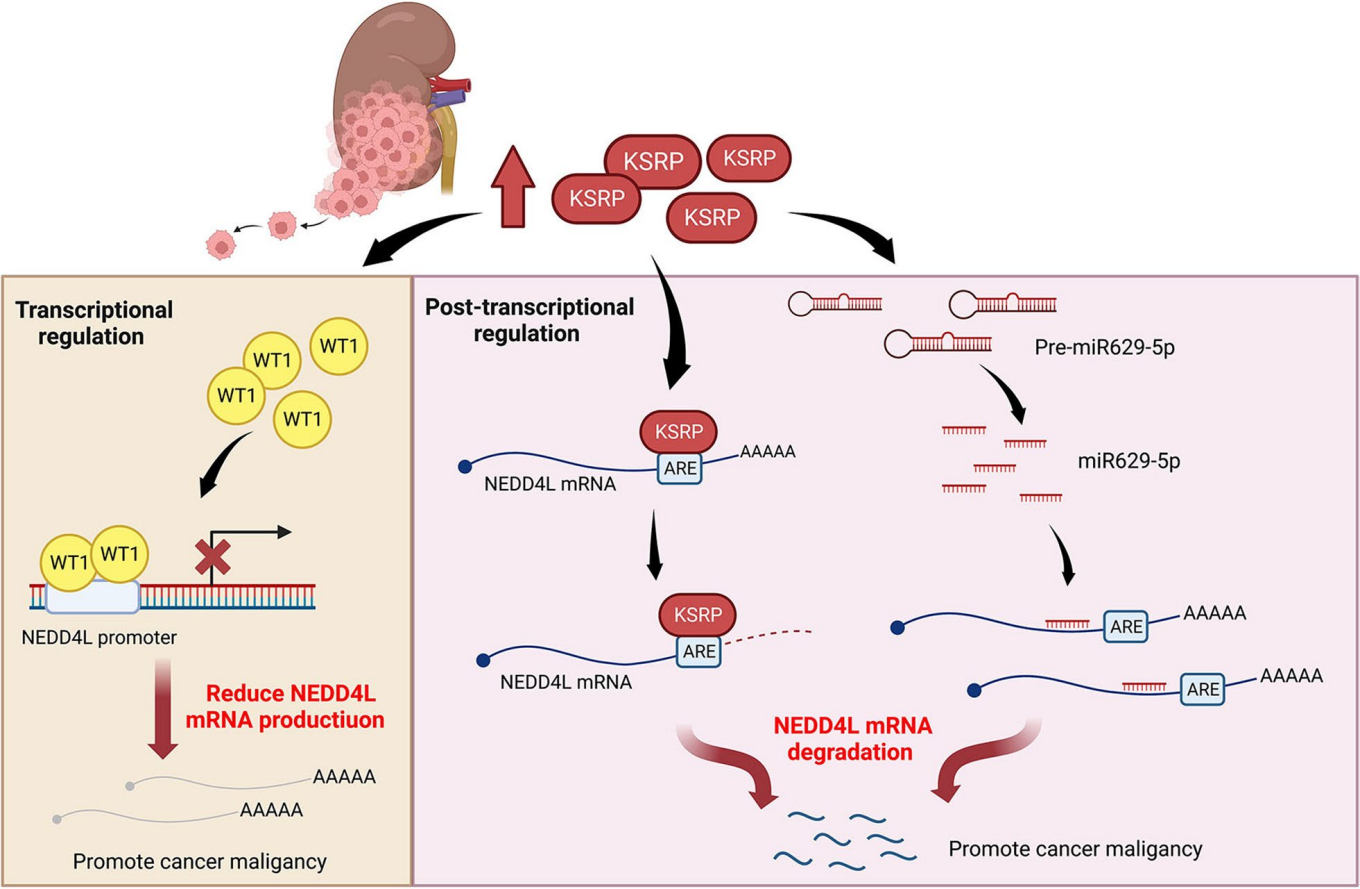
Schematic presentation depicting the mechanisms of KH-type splicing regulatory protein (KSRP) in promoting the progression of clear cell renal cell carcinoma (ccRCC) via targeting neural precursor cell-expressed developmentally downregulated 4 like (NEDD4L) (Created with BioRender.com)

In summary, the present study has demonstrated that KSRP acts as a tumor promoter in ccRCC, and NEDD4L is a critical determinant and is negatively regulated by KSRP to execute its prometastatic effect via EMT induction. Mechanistic investigations showed that NEDD4L may negatively regulate EMT via degrading upstream activators of the PI3K/Akt pathway. We further identified novel mechanisms of KSRP in modulating NEDD4L gene expression via post-transcriptional and transcriptional mechanisms including increases in miR-629-5p induction, NEDD4L ARE binding, and WT1 upregulation (Fig. 7). The identified oncogenic roles of KSRP and miR-629-5p in ccRCC present promising opportunities for the development of new therapies. Exploring the design of agents that can specifically target the active domain of KSRP or block the function of miR-629-5p in a highly specific manner in ccRCC is an avenue worth investigating. In particular, considering the consistent pro-tumoral role of miR-629-5p that has been observed in various cancers in recent years [30, 41], targeting miR-629-5p may be a more promising approach for therapeutic applications in cancer. This approach could potentially offer advantages such as fewer side effects compared to the conflicting discoveries related to KSRP's role in cancer. Further research and development in this area could lead to novel and targeted therapeutic strategies for ccRCC and other cancers.

### Supplementary Information

**Additional file 1: Figure S1.** Violin plot indicates that KSRP expression was elevated in metastatic and non-metastatic ccRCC tumor tissues compared to normal tissues. **Figure S2.** Overexpression of KH-type splicing regulatory protein (KSRP) promotes invasion of clear cell renal cell carcinoma (ccRCC) cells. **Figure S3.** Effect of KH-type splicing regulatory protein (KSRP)-knockdown on neural precursor cell-expressed developmentally downregulated 4 like (NEDD4L) expression in papillary renal cell carcinoma (pRCC) cells. **Figure S4.** Violin plot indicating that NEDD4L expression was decreased in metastatic and non-metastatic ccRCC tumor tissues compared to normal tissues. **Figure S5.** Effect of NEDD4L on cell invasion ability in 786-O cells. **Figure S6.** Inhibition of NEDD4L restored the invasion ability via KSRP-KD in 786-O and ACHN cells. **Figure S7.** Effect on the mesenchymal or epithelial markers by KSRP-KD or NEDD4L overexpression in 786-O cells. **Figure S8.** Flowchart indicates the analysis pipeline to identify NEDD4L’s substrates and those signaling pathways in which these substrates participate. **Figure S9.** p-Akt and Snail are the substrates of NEDD4L that mediate the EMT-related pathway. **Figure S10.** Dissemination and metastasis of ccRCC cells in zebrafish embryos. **Figure S11.** Inhibition of KSRP resulted in the prolongation of NEDD4L mRNA half-life. **Figure S12.** Identification of the key miRNA induced by KSRP in ccRCC. **Figure S13.** KSRP facilitates the biogenesis of has-miR-629-5p in ccRCC cells. **Figure S14.** Effect of miR-629-5p on NEDD4L expression and invasive ability. **Figure S15.** The investigation of a miR-629-5p inhibitor on KSRP-induced downregulation of NEDD4L and promotion of invasion. **Figure S16.** Elevation of miR-629-5p in the tissues with higher malignancy. **Figure S17.** Prognostic effect of miR-629-5p in patients with clear cell renal cell carcinoma (ccRCC). **Figure S18.** Effect of the mutations on ARE sites of NEDD4L mRNA. **Figure S19.** Luciferase reporter activity driven by different NEDD4L promoter segments in 786-O cells with or without knocking down KSRP. **Figure S20.** Effects of KSRP on Wilm's tumor 1 (WT1) expression in clear cell renal cell carcinoma (ccRCC) cells.

## Data Availability

All data generated or analyzed during this study are included in this published article and its additional files. The data used in the current study are available from the corresponding authors upon reasonable request.

## Abbreviations

ARE: AU-rich element
ccRCC: Clear cell renal cell carcinoma
cDNA: Complementary DNA
DSS: Disease-specific survival
EMT: Epithelial–mesenchymal transition
FC: Fold change
FDR: False discovery rate
GEO: Gene Expression Omnibus
GSEA: Gene set enrichment analysis
IHC: Immunohistochemistry
KSRP: KH-type splicing regulatory protein
miRNA/miR: MicroRNA
NEDD4L: Neural precursor cell-expressed developmentally downregulated 4 like
NES: Normalized enrichment score
OS: Overall survival
RBP: RNA-binding protein
RPM: Reads per million mapped reads
RT-qPCR: Reverse-transcription quantitative polymerase chain reaction
TCGA-KIRC: The Cancer Genome Atlas Kidney Renal Clear Cell Carcinoma
TF: Transcription factor
3’UTR: 3-Untranslated region
TMA: Tissue microarray
WT1: Wilms’ tumor 1
