# Supplementary material for: The RNA-binding protein KSRP aggravates malignant progression of clear cell renal cell carcinoma through transcriptional inhibition and post-transcriptional destabilization of the NEDD4L ubiquitin ligase
Source: J Biomed Sci. 2023 Aug 14;30:68. doi: 10.1186/s12929-023-00949-9 (PMC10424398; doi:10.1186/s12929-023-00949-9)
Supplement: Supplementary file 1 — Additional file 1: Figure S1. Violin plot indicates that KSRP expression was elevated in metastatic and non-metastatic ccRCC tumor tissues compared to normal tissues. Figure S2. Overexpression of KH-type splicing regulatory protein (KSRP) promotes invasion of clear cell renal cell carcinoma (ccRCC) cells. Figure S3. Effect of KH-type splicing regulatory protein (KSRP)-knockdown on neural precursor cell-expressed developmentally downregulated 4 like (NEDD4L) expression in papillary renal cell carcinoma (pRCC) cells. Figure S4. Violin plot indicating that NEDD4L expression was decreased in metastatic and non-metastatic ccRCC tumor tissues compared to normal tissues. Figure S5. Effect of NEDD4L on cell invasion ability in 786-O cells. Figure S6. Inhibition of NEDD4L restored the invasion ability via KSRP-KD in 786-O and ACHN cells. Figure S7. Effect on the mesenchymal or epithelial markers by KSRP-KD or NEDD4L overexpression in 786-O cells. Figure S8. Flowchart indicates the analysis pipeline to identify NEDD4L’s substrates and those signaling pathways in which these substrates participate. Figure S9. p-Akt and Snail are the substrates of NEDD4L that mediate the EMT-related pathway. Figure S10. Dissemination and metastasis of ccRCC cells in zebrafish embryos. Figure S11. Inhibition of KSRP resulted in the prolongation of NEDD4L mRNA half-life. Figure S12. Identification of the key miRNA induced by KSRP in ccRCC. Figure S13. KSRP facilitates the biogenesis of has-miR-629-5p in ccRCC cells. Figure S14. Effect of miR-629-5p on NEDD4L expression and invasive ability. Figure S15. The investigation of a miR-629-5p inhibitor on KSRP-induced downregulation of NEDD4L and promotion of invasion. Figure S16. Elevation of miR-629-5p in the tissues with higher malignancy. Figure S17. Prognostic effect of miR-629-5p in patients with clear cell renal cell carcinoma (ccRCC). Figure S18. Effect of the mutations on ARE sites of NEDD4L mRNA. Figure S19. Luciferase reporter activity driven [file 12929_2023_949_MOESM1_ESM.pdf]

Supplemental Information

Title:

The RNA-binding protein KSRP aggravates malignant progression of clear cell renal cell carcinoma through transcriptional inhibition and post-transcriptional destabilization of the NEDD4L ubiquitin ligase

Yi-Chieh Yang, Yung-Wei Lin, Wei-Jiunn Lee, Feng-Ru Lai, Kuo-Hao Ho, Chih-Ying Chu, Kuo-Tai Hua, Ji-Qing Chen, Min-Che Tung, Michael Hsiao, Yu-Ching Wen, Ming-Hsien Chien

Correspondence to: Dr. Yu-Ching Wen (E-mail: 95207@w.tmu.edu.tw) and Dr. Ming-Hsien Chien (E-mail: mhchien1976@gmail.com)

Figure Legends

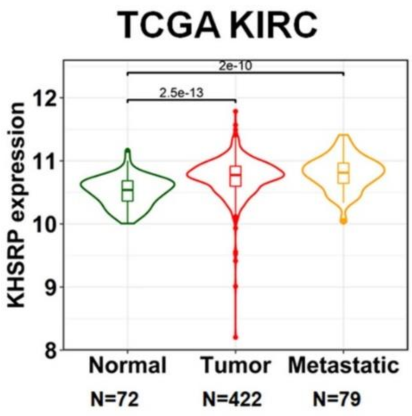

Figure S1. Violin plot indicates that KH-type splicing regulatory protein (KSRP) expression was elevated in metastatic and non-metastatic clear cell renal cell carcinoma (ccRCC) tumor tissues compared to normal tissues. A Kruskal-Wallis test with Dunn's post-hoc test was performed to compare KSRP levels among normal (*n*=72), tumor (*n*=422), and metastatic tissues (*n*=79).

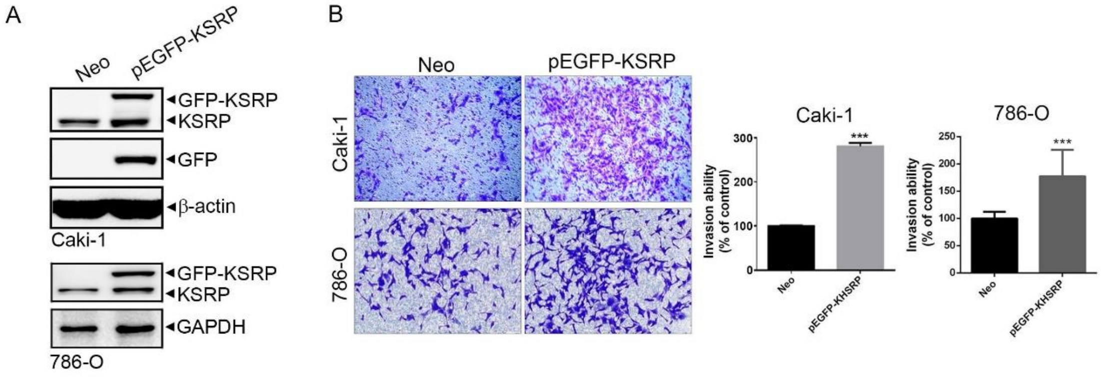

Figure S2. Overexpression of KH-type splicing regulatory protein (KSRP) promotes invasion of clear cell renal cell carcinoma (ccRCC) cells. Caki-1 and 786-O ccRCC cells were transfected with control vector or pEGFP-KSRP. Expression of KSRP and invasive abilities of cells were respectively determined through Western blot (A) and Matrigel invasion (B) assays. Values are presented as the mean ± SD of three independent experiments. *** $p < 0.001$, compared to the control group.

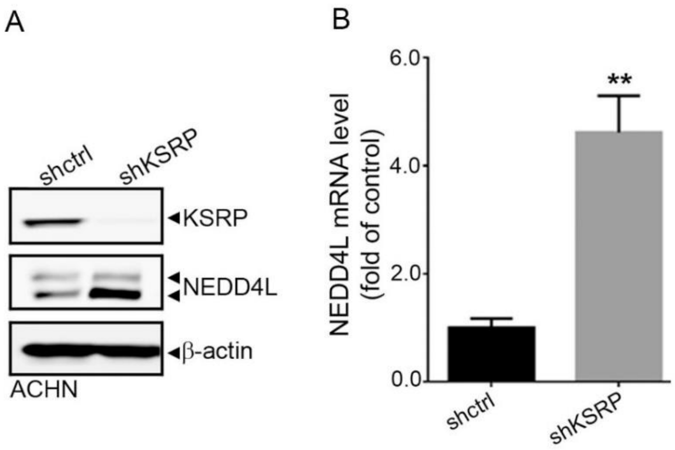

Figure S3. Effect of KH-type splicing regulatory protein (KSRP)-knockdown on neural precursor cell-expressed developmentally downregulated 4 like (NEDD4L) expression in papillary renal cell carcinoma (pRCC) cells. Western blotting (A) and real-time qPCR (B) analyses of NEDD4L levels in ACHN pRCC cells transfected with the KSRP shRNA or shCtrl. Values are presented as the mean ± SD of three independent experiments. ** $p < 0.01$, compared to the control group.

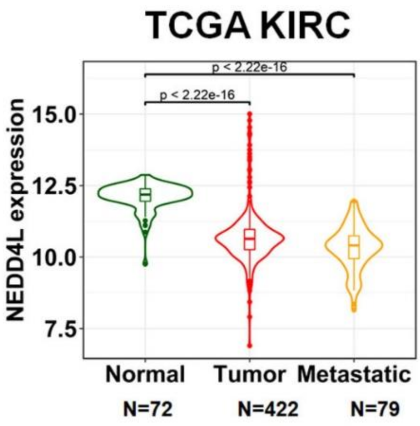

Figure S4. Violin plot indicating that neural precursor cell-expressed developmentally downregulated 4 like (NEDD4L) expression was decreased in metastatic and non-metastatic clear cell renal cell carcinoma (ccRCC) tumor tissues compared to normal tissues. A Kruskal-Wallis test with Dunn's post-hoc test was performed to compared NEDD4L levels among normal (*n*=72), tumor (*n*=422), and metastatic tissues (*n*=79).

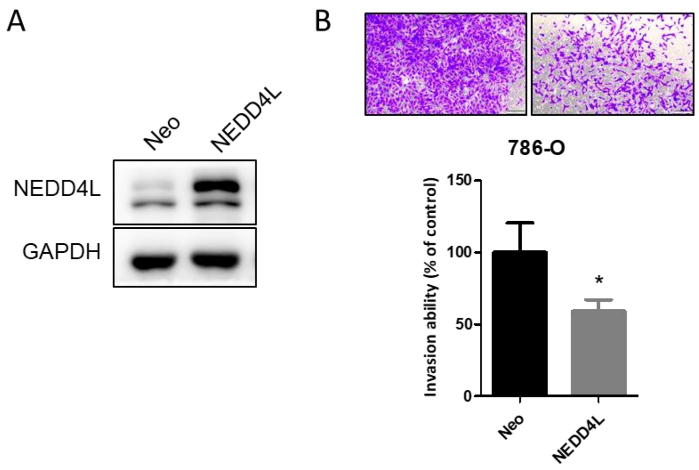

Figure S5. Effect of NEDD4L on cell invasion ability in 786-O cells. The expression of NEDD4L and invasive abilities of cells were respectively determined through Western blot (A) and Matrigel invasion (B) assays. Values are presented as the mean ± SD of three independent experiments. * $p < 0.05$, compared to the control group.

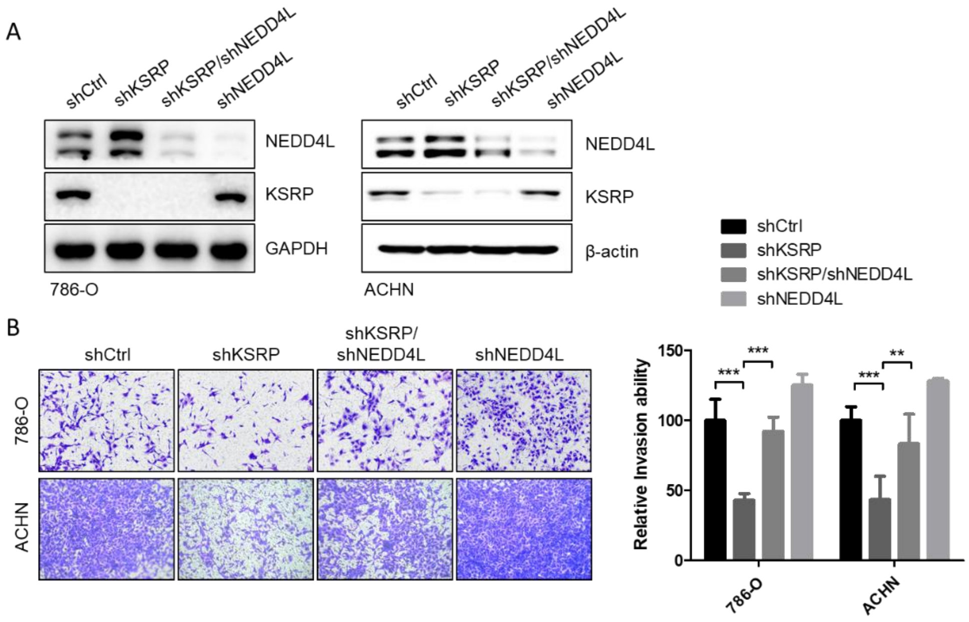

Figure S6. Inhibition of NEDD4L restored the invasion ability via KSRP-KD in 786-O and ACHN cells. The invasion ability was restored by inhibiting NEDD4L in 786-O and ACHN cells via knockdown of KSRP. The cells were infected with shCtrl, shKSRP, shNEDD4L, or shKSRP/shNEDD4L. The expression levels of KSRP and NEDD4L, as well as the invasive abilities of the cells, were determined using Western

blot (A) and Matrigel invasion (B) assays. ** $p < 0.01$; *** $p < 0.001$, compared to the shKSRP group.

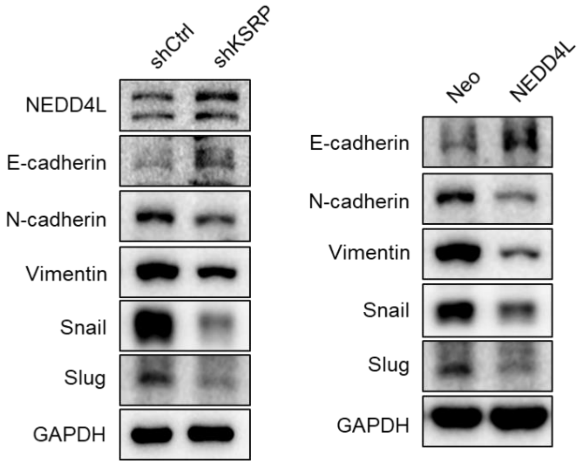

Figure S7. Effect on the mesenchymal or epithelial markers by KSRP-KD or NEDD4L overexpression in 786-O cells. Western blotting analyses of mesenchymal (N-Cadherin, Vimentin, Snail, and Slug) and epithelial (E-cadherin) marker expression in 786-O cells with the KSRP-KD or NEDD4L-overexpressed.

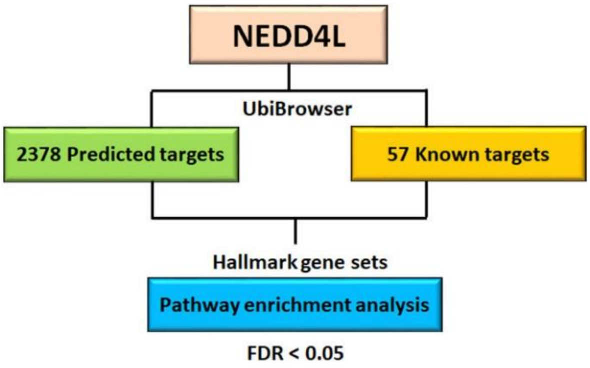

Figure S8. Flow chart indicating the analysis pipeline to identify the potential substrates of neural precursor cell-expressed developmentally downregulated 4 like (NEDD4L) and in which signaling pathways these substrates participate. UbiBrowser (http://ubibrowser.ncpsb.org/ubibrowser/home/index) was used to predict the potential substrates of NEDD4L, and 57 known and 2378 predicted substrates were found. A pathway enrichment analysis was further performed to investigate the top signaling pathways involved in potential NEDD4L substrates.

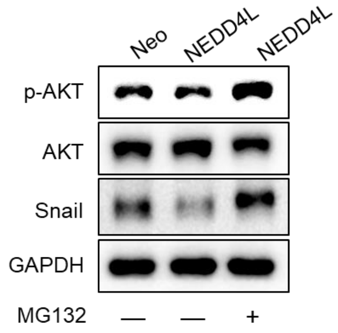

Figure S9. p-Akt and Snail are the substrates of NEDD4L that mediate the EMT-related pathway. 786-O cells with NEDD4L overexpressed were treated with or without 20 μM MG-132 for 4 h, and then the Akt, p-Akt, Snail, and GAPDH proteins were analyzed by Western Blot analysis.

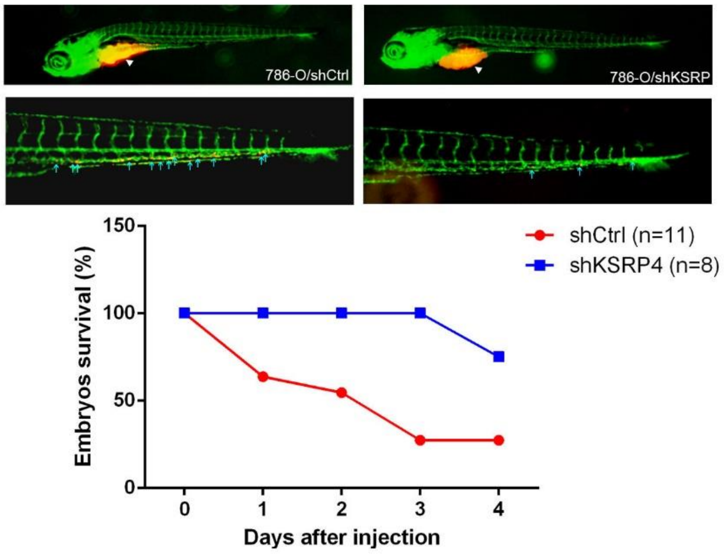

Figure S10. Dissemination and metastasis of 786-O clear cell renal cell carcinoma (ccRCC) cells in zebrafish embryos. 786-O cells were implanted into 48 h post-fertilization zebrafish embryos. Tumor cell dissemination and metastasis were detected at day 3 post-injection. White arrows indicate the primary tumor, and blue arrowheads indicate disseminated tumor foci. Lower panel, Survival curve of zebrafish embryos injected with 786-O/shKSRP or 786-O/shCtrl cells.

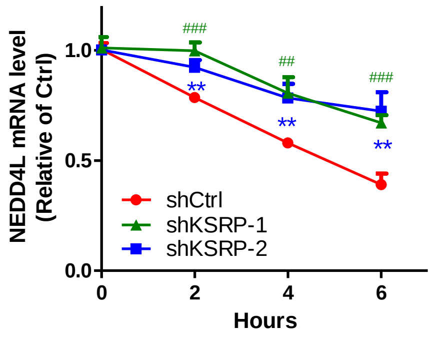

Figure S11. Inhibition of KSRP resulted in the prolongation of NEDD4L mRNA half-life. To examine the decay rate of NEDD4L mRNA, 786-O cells were treated with 5 μg/ml actinomycin D at indicated time points, with or without knocking down KSRP.

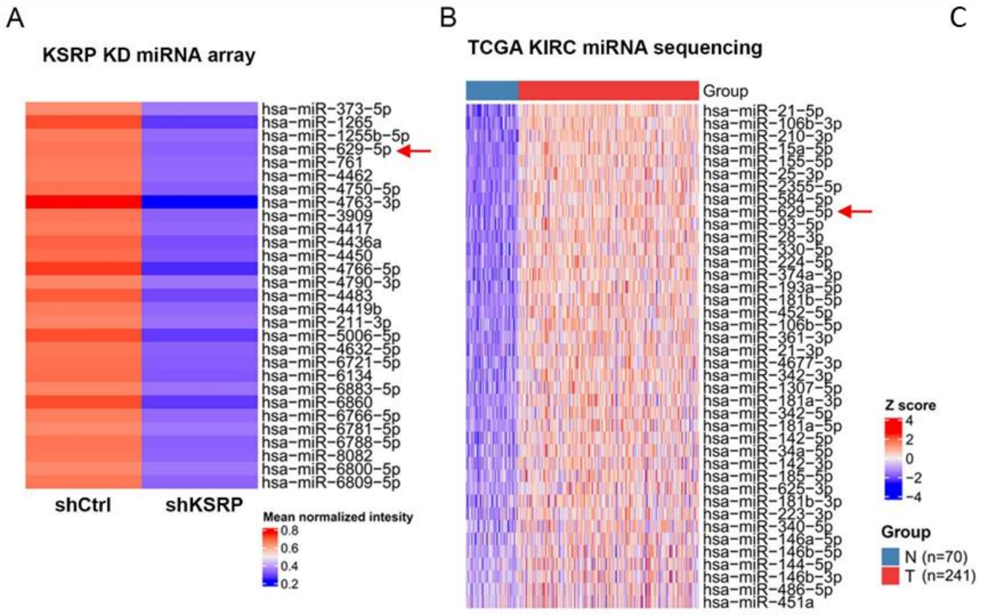

Figure S12. Identification of the key miRNA induced by KH-type splicing regulatory protein (KSRP) in clear cell renal cell carcinoma (ccRCC). (A) miRNAs exhibited a fold change (FC) of < 0.5 in KSRP-depleted ccRCC cells compared to control cells as shown in a heatmap. (B) miRNAs that exhibited an FC of > 2 and a false discovery rate (FDR) of < 0.01 in ccRCC tumor tissues (*n*=241) compared to normal tissues (*n*=70) as shown in the heatmap. The red arrowhead indicates that miR-629-5p was the overlapping miRNA of these two heatmaps.

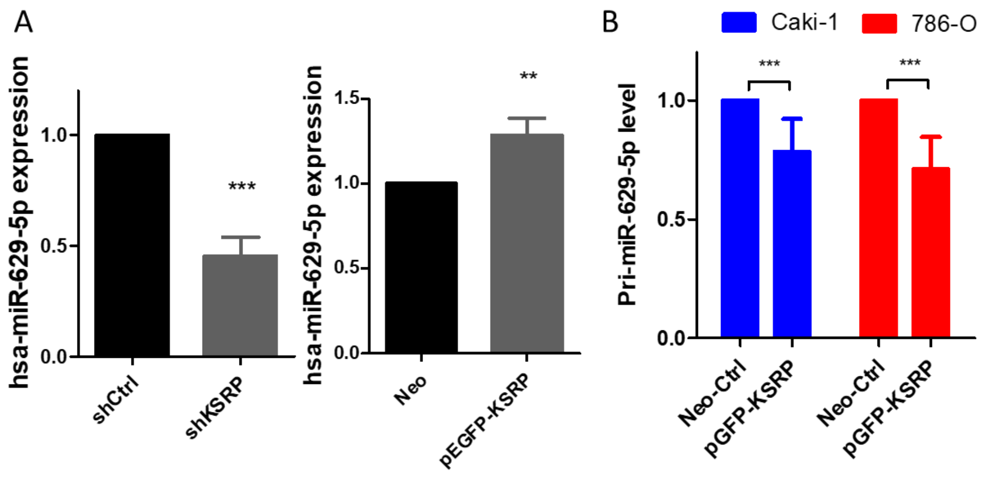

Figure S13. KSRP facilitates the biogenesis of has-miR-629-5p in ccRCC cells. Real-time qPCR analysis of (A) mature miR-629-5p level in 786-O cells and (B) primary miR-629-5p level in ccRCC cells transfected with indicated shKSPR, pEGFP-KSRP, or control vectors. ** $p < 0.01$; *** $p < 0.001$, compared to the control group.

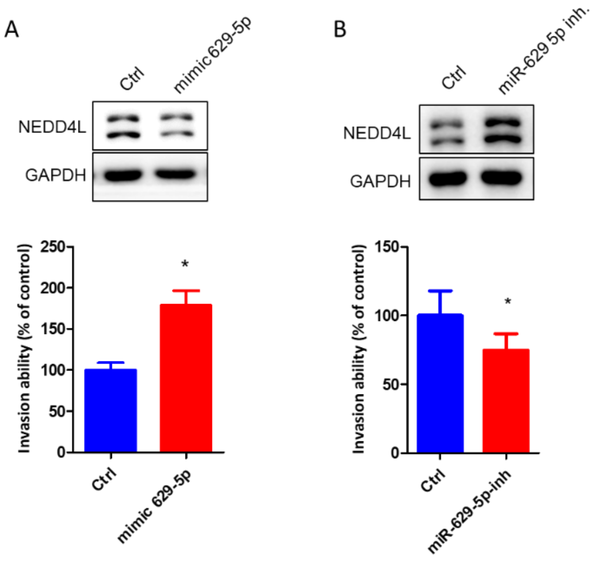

Figure S14. Effect of miR-629-5p on NEDD4L expression and invasive ability. 786-O cells were transfected with a miR-629-5p mimic (A) or miR-629-5p inhibitor (B) for 24 h. NEDD4L protein levels and invasive ability of cells were respectively determined by Western blot (WB) (up panel) and Matrigel invasion (low panel) assays. * $p < 0.05$, compared to the control group.

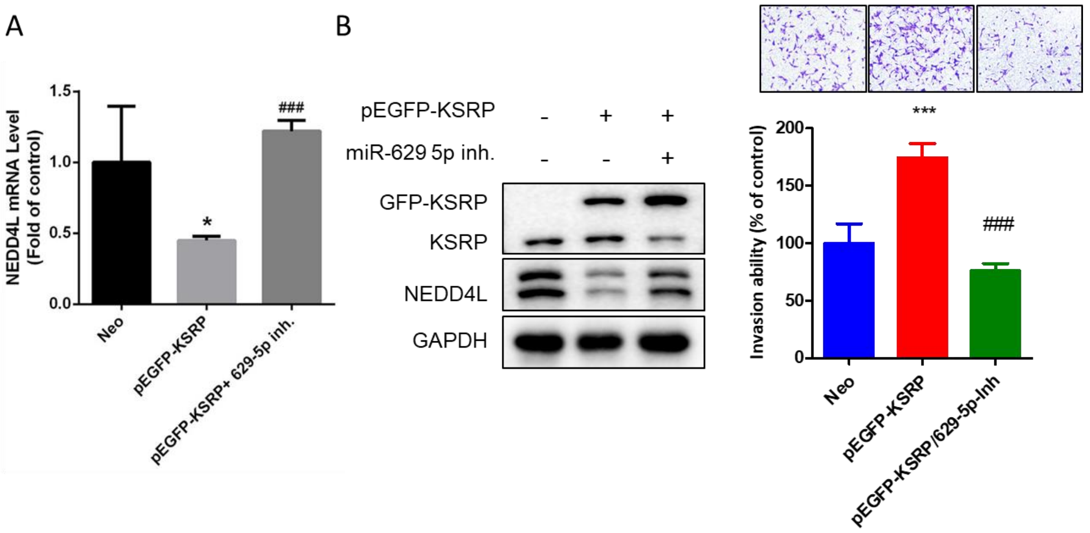

Figure S15. The investigation of a miR-629-5p inhibitor on KSRP-induced downregulation of NEDD4L and promotion of invasion. (A) Real-time qPCR analysis of NEDD4L levels in Caki-1 cells transfected with the pEGFP-KSRP with or without an miR-629-5p inhibitor. (B) Determining of KSRP and NEDD4L protein level (left panel) and invasive ability of cells (right panel) in 786-O cells transfected with pEGFP-KSRP with or without an miR-629-5p inhibitor. Values are presented as the mean ± SD of three independent experiments. * $p < 0.05$, *** $p < 0.001$, compared to the control group; ### $p < 0.001$, compared to the KSRP-overexpressing group.

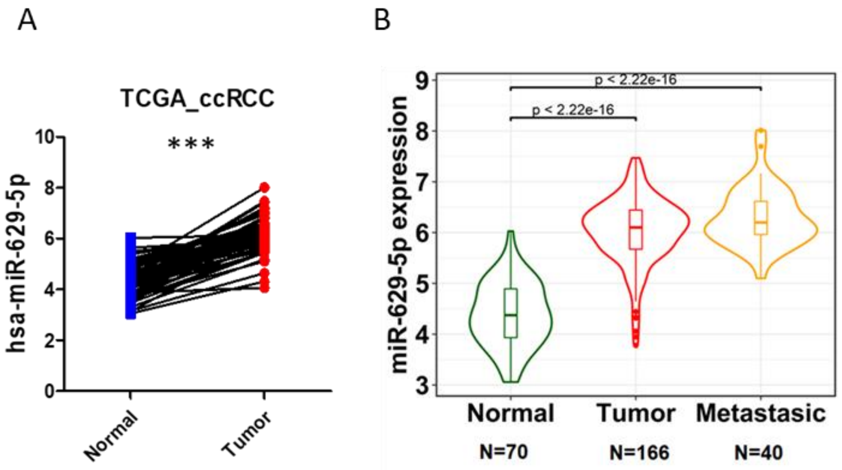

Figure S16. Elevation of miR-629-5p in the tissues with higher malignancy. (A) Elevated miR-629-5p in the paired cancerous tissues compared to adjacent normal tissues from the TCGA ccRCC cohort (n=67). (B) Violin plot demonstrating that miR-629-5p expression was elevated in metastatic and non-metastatic ccRCC tumor tissues

compared to normal tissues. A Kruskal-Wallis test with Dunn's post-hoc test was performed to compare miR-629-5p levels among normal (*n*=70), tumor (*n*=166), and metastatic tissues (*n*=40).

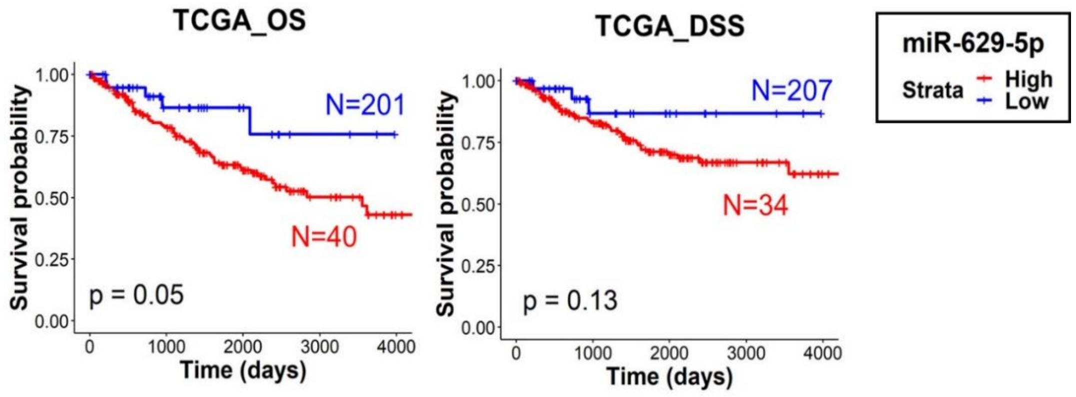

Figure S17. Prognostic effect of miR-629-5p in patients with clear cell renal cell carcinoma (ccRCC). Kaplan-Meier analysis of overall survival (OS) and disease-specific survival (DSS) rates in patients with ccRCC presenting with high or low expression of miR-629-5p using data from TCGA-KIRC.

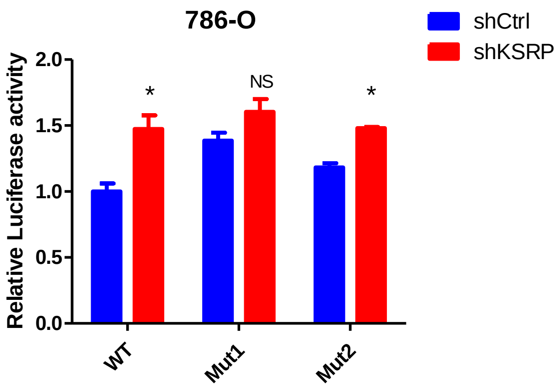

Figure S18. Effect of the mutations on ARE sites of NEDD4L mRNA. 786-O cells with or without knocking down KSRP transfected with the NEDD4L luciferase 3'-UTR reporter vector containing wild-type or mutant ARE domains. * $p < 0.05$, compared to the respective control groups.

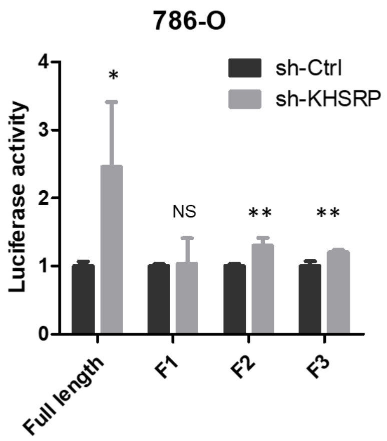

Figure S19. Luciferase reporter activity driven by different NEDD4L promoter segments was assessed in 786-O cells with or without knocking down KSRP.

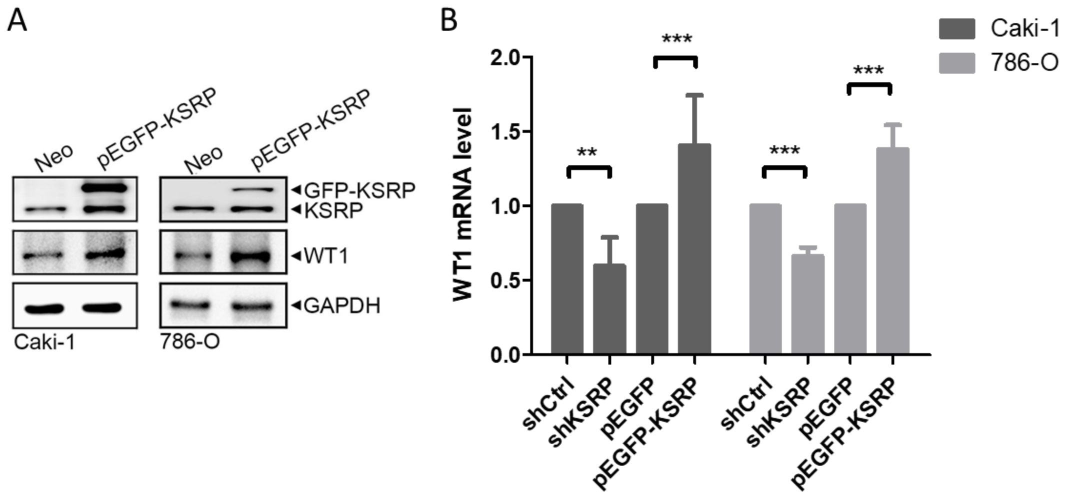

Figure S20. Effects of KSRP on Wilm's tumor 1 (WT1) expression in clear cell renal cell carcinoma (ccRCC) cells. The expression level of WT1 was examined using Western blot analysis (A) and q-PCR assay (B) in Caki-1 and 786-O cells with manipulation of shKSRP or pEGFP-KSRP.
